# Interactions of PGPR from the phylum bacillota with native rhizosphere microbiota: current insights and future perspectives

**DOI:** 10.1007/s11274-026-05007-8

**Published:** 2026-05-08

**Authors:** Magdalena Szpytma, Jakub Dobrzyński

**Affiliations:** Department of Agricultural and Environmental Microbiology, Institute of Technology and Life Sciences - National Research Institute, Al. Hrabska 3, Raszyn, 05-090 Poland

**Keywords:** Plant growth-promoting rhizobacteria, Bacillota, Rhizosphere microbiome, Microbiome modulation

## Abstract

The intensive use of synthetic fertilizers and pesticides has increased crop productivity but also contributed to soil degradation and biodiversity loss, highlighting the need for more sustainable agricultural strategies. Among emerging solutions, plant growth-promoting rhizobacteria (PGPR), particularly members of the Bacillota phylum, are gaining attention as effective bioinoculants that enhance plant growth and tolerance to biotic and abiotic stresses. However, introduced strains do not function in isolation. They enter complex microbial communities, shaped by plant type and developmental stage, influenced by soil properties and environmental conditions. While the positive effects of PGPR on plant performance are well documented, their impact on indigenous rhizosphere microbiota remains less studied. This review synthesizes current knowledge on how Bacillota-based inoculants influence native microbial communities in cereals, vegetables, orchard crops, and fiber plants. Most studies report shifts toward plant-beneficial taxa and reduced abundance of potential pathogens following Bacillota application. Frequently enriched genera include *Bacillus*, *Pseudomonas*, *Lysobacter*, *Sphingomonas*, *Streptomyces*, *Azotobacter*, *Arthrobacter*, *Pseudarthrobacter*, *Bradyrhizobium*, *Devosia*, *Flavobacterium*, *Klebsiella*, *Herbaspirillum*, and *Rhodanobacter*. These changes are often associated with improved plant growth and yield, and stress resilience. However, responses strongly depend on strain, plant and methodological approach. We summarize commonly applied approaches used to assess these interactions. Despite technological advances, limitations remain, such as single time-point sampling, simplified experimental systems, and insufficient integration of inoculant persistence with community analyses. Standardized, multi-site experimental frameworks, with multiple sampling terms are needed to improve predictability and ensure the safe implementation of PGPR-based solutions in sustainable agriculture.

## Introduction

The global population is increasing at an unusual rate, driving a continuously rising demand for food production. Enhancement of agricultural productivity often relied on the intensive use of synthetic fertilizers, pesticides and large-scale monocultures. However, these practices contribute to soil degradation, biodiversity loss, and increase of climate changes (Tripathi et al. 2020; Pereira et al. 2025). Additionally, soil erosion and climate-related pressures intensify abiotic stress, in consequence reducing both the quantity and quality of crop yields (2021; Poria et al. 2022; Ocwa et al. 2023; Hussen et al. 2026). In response to these challenges, there is a growing pressure on development of sustainable and environmentally friendly strategies that can reduce overusing inorganic fertilizers and enhance plant resilience to biotic and abiotic stress. Among the promising approaches, gaining increasing attention, is the application of plant growth-promoting rhizobacteria (PGPR), which offer a potential pathway toward more sustainable agricultural systems (Lopes et al. 2021; Dobrzyński et al. 2026c).

Rhizobacteria, bacteria inhabiting the rhizosphere, often establish mutualistic interactions with their host plants, contributing to plant growth promotion and suppression of phytopathogens. Increasing evidence suggests that PGPR can enhance the growth and performance of a wide range of host plants across different taxonomic groups (Khoso et al. 2024; Naziębło et al. 2025). Among the most promising and most studied rhizobacteria genera are *Pseudomonas*, *Bacillus*, *Paenibacillus*, *Enterobacter*, *Serratia*, *Burkholderia*, *Azotobacter*, *Acinetobacter*, *Mesorhizobium*, *Rhizobium*, *Arthrobacter*, and *Klebsiella* (including *Raoultella*) (Li et al. 2022; Dobrzyński and Jakubowska 2025; Dobrzyński et al. 2026b). *Bacillus* and *Paenibacillus* are considered particularly promising as eco-friendly alternatives, due to their biocontrol properties and ability to form resilient endospores that increase survival chances under diverse environmental conditions (Radhakrishnan et al. 2017; Tariq et al. 2025).

The number of studies investigating beneficial effects of PGPR on various plant species increased each year. However, the majority of this research has primarily focused on direct plant-bacteria interactions, while the impact of inoculants on the native rhizosphere microbiota has often been overlooked (De Andrade et al. 2023; Yang et al. 2024b). In cases where such interactions have been examined, studies frequently lack consistency and are limited to one sampling term, single conditions type, and single soil type (Oulkhir et al. 2025; Patyka et al. 2025). This limitation reduces the likelihood of obtaining reproducible and generalizable results.

Despite inconsistencies across studies, most evidence indicates that PGPR inoculation indeed affects the native microbiota (Mawarda et al. 2020). Understanding the interactions between native microbial communities and introduced inoculants is essential for optimizing the effectiveness and ensuring safety of PGPR applications in agriculture (Berg et al. 2021). PGPR inoculants often have problems with surviving in soil, which can reduce their overall effectiveness. Consequently, there is a growing interest in using PGPR not only as direct plant growth enhancers but also as modulators of the native rhizosphere microbiota, which plays crucial role in maintaining soil homeostasis and plant health (Orozco-Mosqueda et al. 2018; Vuolo et al. 2022; Dobrzyński et al. 2026d).

The aim of this review is to summarize the current state of knowledge, identify existing gaps and methodological limitations in assessing the impact of PGPR from Bacillota on native microbial communities, and highlight emerging approaches that may provide more comprehensive insights into these complex interactions.

## Plant growth-promoting rhizobacteria interaction

**Fig. 1:**
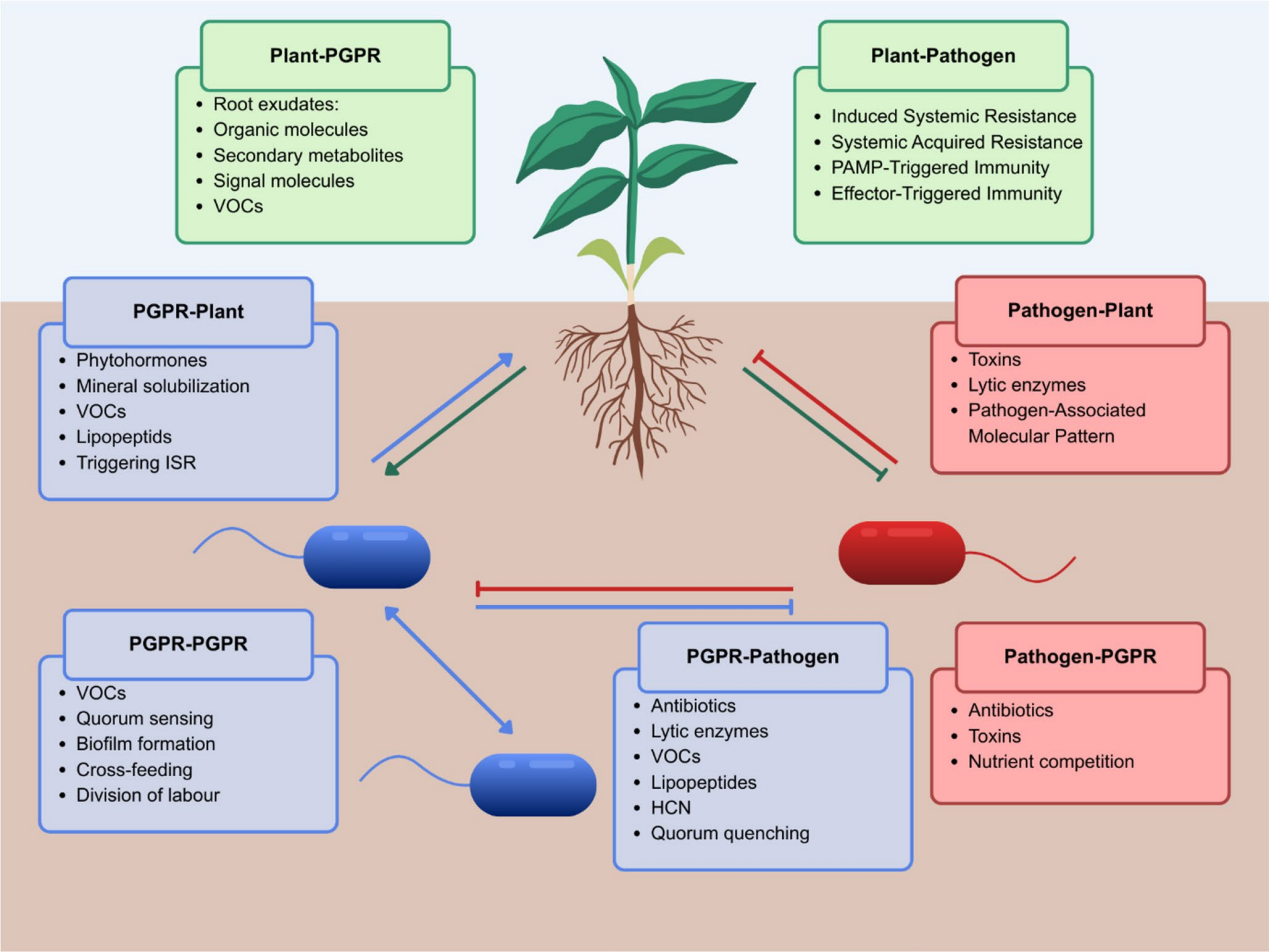
Overview of interaction in the rhizosphere amongst PGPR, host plant, and pathogens (Laishram et al. 2025; Simas et al. 2025; Sibanyoni et al. 2026)

Soil directly surrounding plant roots, called rhizosphere, is characterized by greater microbial abundance and decreased taxonomic diversity compared to bulk soil. This enrichment is primarily driven by root exudates released by plants, which increase the availability of the organic carbon in the soil (Bakker et al. 2013; Sasse et al. 2018; Pathan et al. 2020). Easily available organic compounds make the rhizosphere an attractive niche for a wide range of micro- and macroorganisms, including viruses, bacteria, archaea, fungi, and even other plants and animals. These organisms interact in diverse ways, which can be broadly categorized as antagonistic (e.g., predation, parasitism, or competition) or non-antagonistic (e.g., mutualism or symbiosis) (Leach et al. 2017; Yang et al. 2024a). Rhizobacteria interact both with their host plants and with other members of the microbial community (Fig. 1). The outcomes of these interactions play a crucial role in shaping microbiome structure and directly influence plant health (Chepsergon and Moleleki 2023).

### PGPR interaction with host plants

Rhizosphere bacteria frequently establish mutualistic interactions with plants (Khoso et al. 2024). They protect the host from pathogens, for instance by competing with other microorganisms, including bacteria and fungi, and simultaneously promote plant growth through the production of metabolites and enzymes. Among them, plant growth-promoting rhizobacteria enhance plant development via both direct and indirect mechanisms (Chen et al. 2024). Direct effects include secretion of phytohormones (Goud et al. 2025), nitrogen fixation (Kazerooni et al. 2021), and solubilization of essential minerals such as phosphorus and potassium (Wani et al. 2007; Xiang et al. 2023). Indirect mechanisms involve responding to biotic and abiotic stresses, for instance through induction of systemic resistance (ISR) (Fatouros et al. 2018; Dobrzyński et al. 2023), production of fungistatic and antibiotic compounds, such as lipopeptides (Selim et al. 2010; Bouchard-Rochette et al. 2022) and volatile organic compounds (VOCs) (Liu et al. 2008; Raza et al. 2015; Poulaki and Tjamos 2023), and secretion of hydrolytic enzymes that degrade the cell walls of pathogens (Drewnowska et al. 2020). Rhizobacteria can employ multiple growth-promoting mechanisms simultaneously, with their activity depending on environmental conditions and developmental stage of the host plant (Li et al. 2022).

## PGPR interaction with native microbiota

In contrast to the extensively studied interactions between PGPR and host plants, interactions between PGPR and other bacteria in the rhizosphere remain relatively understudied. Most available studies focused primarily on antagonistic relationships between PGPR and plant pathogens. However, to fully use the potential of PGPR inoculants and improve their effectiveness and persistence in soil, it is essential to understand both antagonistic and non-antagonistic interactions between introduced strains and the native microbiota (Berg et al. 2021; Cotta et al. 2025).

Bacteria typically inhabit environments densely populated by numerous species, where intense competition for limited nutrients drives strong selective pressures. Consequently, many bacterial taxa evolved diverse and highly specialized systems for nutrient acquisition, including siderophore production and efficient uptake transporters. In parallel, bacteria frequently synthesize a wide array of antimicrobial compounds that enable them to eliminate competing microorganisms (Chepsergon and Moleleki 2023).

*Bacillus*, *Paenibacillus*, *Priestia* and *Brevibacillus* are among the most extensively studied PGPR genera within the Bacillota phylum. These bacteria are known for producing a broad spectrum of antimicrobial metabolites, including lipopeptides, VOCs, and hydrolytic enzymes. Lipopeptides are cyclic amphiphilic oligopeptides with strong antimicrobial activity, synthesized by numerous *Bacillus* and *Paenibacillus* species. The most prominent classes of these compounds include surfactins, iturins, fengycins and polymyxins (Jiang et al. 2016; Fujita and Yokota 2019; Zhou et al. 2020; Hossain et al. 2023). VOCs are low-molecular-weight chemicals that easily evaporate and disperse under standard temperature and pressure conditions. They are produced by both plants and microorganisms, serving as key mediators of interspecies communication. Microbial VOCs (MVOCs) affect gene expression, membrane permeability, and enzymatic activity in target organisms. Acting as signaling molecules, VOCs may have either positive or negative effects on targeted organisms, by modulating their physiological and behavioral responses (Veselova et al. 2019; Poveda 2021). Some MVOCs exhibit inhibitory or antimicrobial activity against other microorganisms or induce ISR in plants, enhancing their defense against pathogens (Raza et al. 2015; Poulaki and Tjamos 2023). Interestingly, certain bacterial VOCs may stimulate the growth of specific microbial taxa while suppressing others (Garbeva et al. 2014; Kong et al. 2021). Siderophores represent another key class of secondary metabolites produced by rhizosphere bacteria. These iron-chelating compounds are taken up through highly specific receptor-mediated transport systems, allowing bacteria equipped with compatible receptors to access chelated iron. As a result, siderophore production serves as competitive advantage by limiting iron availability to organisms lacking the appropriate uptake mechanisms, while simultaneously facilitating cooperative interactions within closely related species that share similar siderophore receptors (Singh et al. 2022; Chepsergon and Moleleki 2023).

Except for antagonistic interactions, PGPR can also engage in neutral or beneficial interplays with members of native microbiota, contributing to increased microbial diversity and enrichment of beneficial microbials (Mmotla et al. 2025). PGPR inoculation can modify soil physicochemical properties, such as nutrient availability and pH, thereby constructing ecological niches that enhance their own fitness and persistence (Moore et al. 2022). In addition, PGPR can influence rhizosphere functioning by modulating root exudation patterns (Zuluaga et al. 2021). Root exudates serve not only as sources of carbon and nitrogen but also as chemoattractants or chemorepellents, playing a crucial role in microbial colonization and biofilm formation (Mahapatra et al. 2022). Members of dominant PGPR groups, including *Bacillus* spp., are well-known biofilm-forming bacteria. Cooperative biofilm formation represents an important survival strategy that favor PGPR persistence in soil under stress conditions and promotes bacterial communication and cooperation (Ahmad Ansari et al. 2023; Rafique et al. 2024). Yannarell et al. (2019) demonstrated cooperative biofilm formation between *Bacillus subtilis* and *Pantoea agglomerans*, which required *P. agglomerans* exopolysaccharide and the *B. subtilis* amyloid-like protein TasA. Synergistic biofilm formation depending on TasA was later confirmed by Sun et al. (2022) between *Bacillus velezensis* SQR9 and *Pseudomonas stutzeri* XL272. Such interspecies cooperation is hypothesized to be facilitated by exchanging nutrients through cross-feeding. Cross-feeding between *B. velezensis* SQR9 and *Pseudomonas* spp. was further reported by Gu et al. (2025). Another cooperation strategy that is essential for microbiome functioning is the division of labor. Division of labor refers to the specialization of distinct microbial populations in specific steps of a complex metabolic pathway, with the overall process being completed collectively (Zhang et al. 2016). Nitrification is one of fundamental examples of division of labor in the rhizosphere. Nitrification, the oxidation of ammonia (NH_3_) via nitrite (NO^− 2^) to nitrate (NO^− 3^), is usually performed in two steps by ammonia-oxidizing bacteria or archea (AOB, AOA) and then nitrite-oxidizing bacteria (NOB) (Besset-Manzoni et al. 2018).

## Reported impact of Bacillota inoculation on native rhizosphere microbiota

In recent years, an increasing number of studies on PGPR have investigated their effects on the native rhizosphere microbiota. According to Berg et al. (2021), six major types of microbiome modulation can be distinguished: transient microbiome shifts, stabilization or increases in microbial diversity or evenness, restoration of dysbiosis, targeted shifts toward potentially beneficial taxa, and depletion of potential pathogens. This chapter will review the documented impacts of PGPR inoculation from Bacillota phylum on native rhizosphere microbiota of plants relevant to agriculture, horticulture, and fiber production.

## Cereal crops

Several studies have demonstrated that inoculation with bacteria from the Bacillota phylum reshapes structure of the native rhizosphere microbiota, modulates microbial diversity and evenness, and decreases relative abundance of plant pathogens in the rhizosphere of cereal crops. These changes were typically correlated with improvements in biometric parameters and crop yield and quality. A detailed overview of the effect of inoculation with bacteria from the Bacillota phylum on the native microbiome of cereal crops is presented in Table 1.

The most frequently observed effect following inoculation is a shift in the microbiome toward beneficial bacterial groups, including plant growth-promoting taxa. For example, Zhao et al. (2023) reported that inoculation of maize with *B. subtilis* NCD-2 led to an increased relative abundance of genera associated with plant growth promotion, including *Azotobacter*, *Kribbella*, *Rhodococcus*, and *Actinoplanes*. Likewise, Li et al. (2021b) reported enrichment of several genera linked to plant health, such as *Bacillus*, *Pseudomonas*, *Salinimicrobium*, *Lysobacter*, *Devosia*, *Klebsiella*, and *Azotobacter*, after inoculation maize with nitrogen-fixing *Paenibacillus triticisoli* BJ-18. Additionally, treatment with *P. triticisoli* BJ-18 decreased relative abundance of potential fungal pathogens (*Fusarium* and *Gibberella*). Similar trends were observed following the application of *B. velezensis* BS89 and *B. megatrium* OQ560352, where PGPR treatments also promoted a shift toward plant-beneficial bacterial groups (Chebotar et al. 2023; Zhu et al. 2023).

Corresponding pattern was demonstrated by Patyka et al. (2025) in winter wheat following treatment with *B. subtilis* H38. Inoculation increased bacterial diversity and evenness, while enriching plant-growth promoting genera, such as *Bacillus*, *Pseudomonas*, *Azotobacter*, *Streptomyces*, and *Arthrobacter*. Similarly, Li et al. (2021c) reported increased abundance of *Bacillus* and *Pseudomonas* and other beneficial genera (*Pseudarthrobacter*, *Azospirillum*, *Klebsiella*, and *Paenibacillus*) after application with *P. triticisoli* BJ-18. Likewise, Oulkhir et al. (2025) observed increased relative abundance of genera known for their roles in nitrogen fixation and phytohormone production, such as *Paramesorhizobium*, *Ensifer*, and *Hoeflea* in durum wheat inoculated with *B. subtilis* ED24.

In rice Win et al. (2020) noted an increase of bacterial diversity after treatment with *B. pumilus* TUAT-1. Enrichment of beneficial bacterial taxa was reported by Ye et al. (2024) after inoculation of *Priestia megaterium* YB-3. Correspondingly, Chang et al. (2024) observed enrichment of several plant-growth promoting genera, for example *Sphingomonas*, *Flavobacterium*, *Devosia*, *Rhizobacter*, *Herbaspirillum*, and *Azospirillum*, after application with *Bacillus altitudinis* LZP02. Ali et al. (2025) demonstrated depletion of pathogenic fungi and restoration of dysbiosis caused by pathogens, after treatment with *B. velezensis* FZB42.

**Table 1:** Reported impact of Bacillota inoculation on the rhizosphere microbiota of cereal crops. Types of microbiome modulation described by Berg et al. (2021): (A) transient microbiome shifts, (B) stabilization or increase of microbial diversity, (C) stabilization or increase of microbiome evenness, (D) restoration of a dysbiosis/compensation or reduction of a pathogen-induced shift, (E) targeted shifts toward potential beneficial phyla, and (F) depletion of potential pathogens

Inoculant strain(s)	PGP traits	Plant	Type of experiments	Sampling	Impact detection method	Microbiome response	Microbiome modulation type	Reference
*Paenibacillus triticisoli* BJ-18	**Nutrient availability**: N_2_-fixing, siderophores production; **Plant hormone modulation**: IAA and IPA production	Maize	Greenhouse experiment	35 days after transplanting	qPCR − 16 S rRNA, ITS, total diazotrophic *nifH* and *P. triticisoli* BJ-18 *nifH* gene; NGS (Illumina MiSeq) − 16 S rRNA & ITS	**Increase** of potential beneficial bacterial genera (*Bacillus*,* Pseudomonas*,* Salinimicrobium*,* Lysobacter*,* Devosia*, *Klebsiella*, and *Azotobacter*); **Decrease** of potential pathogens (*Fusarium* and *Gibberella*)	E, F	(Li et al. 2021b)
*Bacillus velezensis* BS89	**Antagonistic functions**: hydrolytic enzymes; **Plant hormone modulation**: IAA production	Maize	Field experiment	Before harvest	Pyrosequencing (Roche GSJunior) − 16 S rRNA	**Increase** of potential beneficial bacterial phyla (Verrucomicrobiota, Chloroflexota, Planctomycetota, Pseudomonadota, Bacillota, and Chlamydiota)	E	(Chebotar et al. 2023)
*Bacillus subtilis* NCD-2	**Antagonistic functions**: fengycin and surfactin production; **Activating ISR**	Maize	Pot experiment	35 days after inoculation	NGS (Illumina MiSeq) − 16 S rRNA & ITS; qPCR - strain persistence	**Increase** of Bacteroidota and Gemmatimonadota; **Increase** of potential beneficial bacterial genera (*Kribbella*,* Rhodococcus*,* Actinoplanes*,* Azotobacter*, and *Conexibacter*) **Decrease** of Patescibacteriota, Cyanobacteriota; **Increase** of *Byssochlamys*,* Aspergillus*,* Trichoderma*,* Cephalotrichum*, and *Neurospora*; **Decrease** of *Arthrobotrys*,* Peziza*, and *Simplicillium*	E	(Zhao et al. 2023)
*Bacillus megaterium* OQ560352	Enhancing organic phosphorus utilization	Maize	Greenhouse experiment	after 50 days, at seven-leaf and eleven-leaf stages	NGS (Illumina MiSeq) − 16 S rRNA; WGS (HiSeq 4000)	**Increase** of potential beneficial bacterial phyla (Actinomycetota, Verrucomicrobiota, Bacteroidota, Bacillota, and Mucoromycotina)	E	(Zhu et al. 2023)
*Bacillus subtilis* ED24	**Antagonistic functions**: cellulase production; **Nutrient availability**: siderophores production; **Plant hormone modulation**: ACC deaminase production	Durum wheat	Pot experiment	115 days after sowing	NGS (Illumina MiSeq) − 16 S rRNA & ITS	**Increase** of potential beneficial bacterial genera (*Paramesorhizobium*,* Ensifer*, and *Hoeflea*); **Increase** of potential beneficial fungal genera (*Eurotiomycetes* and *Dothideomycetes*); **Decrease** of potential pathogens (*Sordariomycetes* and *Mycoplasma*)	E, F	(Oulkhir et al. 2025)
*Paenibacillus triticisoli* BJ-18	**Nutrient availability**: N_2_-fixing, siderophores production; **Plant hormone modulation**: IAA and IPA production	Winter wheat	Field experiment	April, May, June	NGS (Illumina MiSeq) − 16 S rRNA & ITS; WGS (HiSeq 2000)	**Increase** bacterial diversity; **Increase** of Bacillota and Planctomycetota; **Increase** of potential beneficial bacterial genera (*Pseudarthrobacter*,* Bacillus*,* Pseudomonas*,* Azospirillum*,* Klebsiella*, and *Paenibacillus*); **Decrease** of potential pathogens (*Alternaria* and *Gibberella*)	B, E, F	(Li et al. 2021c)
*Bacillus subtilis* H38	NA	Winter wheat	Field experiment	Flowering stage	NGS (Illumina MiSeq) − 16 S rRNA; WGS (Illumina NovaSeq 6000); LC-MS/MS	**Increase** bacterial diversity and evenness; **Increase** of potential beneficial bacterial genera (*Bacillus*,* Pseudomonas*,* Azotobacter*,* Streptomyces*, and *Arthrobacter*); **Decrease** of *Sphingomonas* and *Variovorax*	B, C, E	(Patyka et al. 2025)
*Bacillus pumilus* TUAT-1	**Nutrient availability**: siderophores production; **Plant hormone modulation**: IAA production	Rice	Greenhouse experiment	2 and 5 weeks after transplantation	NGS (Illumina MiSeq) − 16 S rRNA; qPCR - strain persistence	**Increase** bacterial diversity; **Increase** of *Desulfuromonadales*; **Decrease** of *Xanthomonadales*	B	(Win et al. 2020)
*Bacillus altitudinis* LZP02	Enhancing carbohydrate metabolism and phenylpropanoid biosynthesis	Rice	Pot experiment	2 weeks after germination	NGS (Illumina MiSeq) − 16 S rRNA & ITS; WGS (HiSeq 2500 PE150)	**Increase** of potential beneficial bacterial genera (*Novosphingobium*,* Acidovorax*,* Sphingomonas*,* Lacibacter*,* Flavobacterium*,* Devosia*,* Rhizobacter*,* Derxia*,* Pelomonas*,* Herbaspirillum*, and *Azospirillum*)	E	(Chang et al. 2024)
*Priestia megaterium* YB-3	Nematicidal properties; **Activating ISR**	Rice	Field experiment	10, 40 and 60 days after treatment	NGS (Illumina MiSeq) − 16 S rRNA & ITS; qPCR - strain persistence; Fluorescent microscope - *gfp* marked strain colonisation assay	**Increase** abundance of Acidobacteriota, Bacteroidota, and Ascomycota	E	(Ye et al. 2024)
*Bacillus velezensis* FZB42	**Antagonistic functions**: fengycin, surfactin, bacillomycin D, macrolactin, bacilysin, difficidin and VOCs production; **Plant hormone modulation**: IAA production **Activating ISR**	Rice	Pot experiment	At the end of the experiment	NGS (Illumina MiSeq) − 16 S rRNA & ITS	**Decrease** of pathogenic fungi	D, F	(Ali et al. 2025)

## Vegetables

Most research on vegetables has shown, similar to findings in cereal crops, a shift toward beneficial microbial groups following inoculation with Bacillota (Table 2). However, some studies have reported only transient changes in the microbial community.

Qiao et al. (2017) demonstrated that application of *B. subtilis* PTS-394 to tomato induced a short-term increase in the relative abundance of several beneficial genera, including *Thermomonas*,* Pseudomonas*,* Lactococcus* and *Rhodanobacter*, while also increasing the overall abundance of the Bacillota phylum. Corresponding pattern was reported by Zhao et al. (2023) following tomato inoculation with *B. subtilis* NCD-2. Treatment enriched well-known beneficial bacterial genera, such as *Bacillus*, *Sphingobium*, and *Ramlibacter*. This type of modulation was also observed after treatment with nitrogen-fixing *B. cereus* DW019 (Dong et al. 2023). Interestingly, Lee Diaz et al. (2024) compared the effects of individual inoculants - *B. amyloliquefaciens*, *Pseudomonas azotoformans*, *Trichoderma harzianum*, and *Rhizophagus irregularis* - with a synthetic community (SynCom) composed of all four strains on the tomato rhizosphere microbiome. Both single bacterial and fungal inoculants, as well as the SynCom, influenced the composition of the rhizosphere microbiome, yet their effects were distinct from each other. Notably, plants inoculated with *B. amyloliquefaciens* CECT8238 or *T. harzianum* T22 exhibited the lowest microbial diversity and the highest number of differentially abundant ASVs compared to other treatments. Despite these differences, all treatments consistently increased the relative abundance of *Ramlibacter*, *Blastococcus* and *Ohtaekwangia*.

Moreover, Kong et al. (2021) demonstrated that tomato inoculation with *B. amyloliquefaciens* GB03 influenced not only the rhizosphere microbiome of the treated plants but also that of neighboring, non-inoculated individuals, effect was mediated by plant-produced VOCs and root exudates.

Qin et al. (2017) reported increment of the relative abundance of *Paenibacillus*, *Rhodanobacter*, and *Pseudomonas* in the cucumber rhizosphere following application with *B. amyloliquefaciens* L-S60. Corresponding results were presented by Shi et al. (2017) after *P. polymyxa* NSY50 treatment. *P. polymyxa* NSY50 application enriched beneficial genera, such as *Bacillus*, *Streptomyces*, and *Pseudomonas*, while decreasing relative abundance of potential fungal pathogens (*Fusarium*). Enrichment of beneficial taxa in cucumber rhizosphere was also observed after inoculation with nitrogen-fixing *B. amyloliquefaciens* FH-1 and *B. subtilis* S1 (Wang et al. 2021; Zhang et al. 2024).

Zhang et al. (2019) reported that treatment with *B. velezensis* NJAU-Z9 increased bacterial diversity and relative abundance of several PGPR-associated genera, including *Sphingomonas*, *Bradyrhizobium*, *Streptomyces*, *Lysobacter*, and *Pseudomonas* in the pepper rhizosphere. Zhao et al. (2023) observed a similar pattern, where *B. subtilis* NCD-2 inoculation also enriched *Bacillus* and *Lysobacter*. Likewise, Liang et al. (2022) reported increased relative abundance of beneficial genera, including *Bacillus*.

As for the lettuce rhizosphere Bai et al. (2024) reported that inoculation with *B. velezensis* SAAS-63 increased relative abundance of *Streptomyces* and *Actinoallomurus* under nutrient deficiency. Correspondingly, Huang et al. (2025) observed enrichment in several beneficial taxa, including *Bacillus* and *Sphingomonas*, following application with phosphate solubilizing *B. amyloliquefaciens* G02.

Application with *Brevibacillus laterosporus* AMCC100017 to potato increased microbial diversity and relative abundance of *Bacillus* and *Pseudomonas* (Chen et al. 2017). Likewise, Li et al. (2021a) reported that treatment with *B. laterosporus* BL12 also enriched *Pseudomonas* and other beneficial genera, such as *Pseudarthrobacter*, *Brevibacillus*, *Massilia*, *Bosea*, and *Devosia*. However, treatment also decreased the relative abundance of *Streptomyces*, *Pedobacter*, and *Terrimonas*, which are considered as plant-beneficial. *B. amyloliquefaciens* QST713 inoculation enriched *Sphingomonas*, while decreasing fungal pathogens (*Fusarium*) (Adamo et al. 2024).

Depletion of fungal pathogens was also reported by Song et al. (2021) after inoculation with *B. subtilis* Bv17. Treatment with *B. subtilis* NCD-2 to eggplant increased relative abundance of *Bacillus*, *Sphingobium*, *Lysobacter*, and *Ramlibacter* (Zhao et al. 2023).

Furthermore, application of *B. subtilis* A-5 and its metabolite γ-PGA to the Chinese cabbage rhizosphere increased the relative abundance of taxa commonly considered beneficial (Bai et al. 2022).

**Table 2:** Reported impact of Bacillota inoculation on the rhizosphere microbiota of vegetables crops. Types of microbiome modulation described by Berg et al. (2021): (A) transient microbiome shifts, (B) stabilization or increase of microbial diversity, (C) stabilization or increase of microbiome evenness, (D) restoration of a dysbiosis/compensation or reduction of a pathogen-induced shift, (E) targeted shifts toward potential beneficial phyla, and (F) depletion of potential pathogens

Inoculant strain(s)	PGP traits	Plant	Type of experiments	Samplig	Impact detection method	Microbiome response	Microbiome modulation type	Reference
*Bacillus subtilis* PTS-394	**Antagonistic functions**: fengycin, iturin and surfactin production	Tomato	Pot experiment	1, 3, 7, 9 and 14 days after treatment	Pyrosequencing (Roche 454 GS FLX) − 16 S rRNA & ITS	Transient microbiome shift: **Increase** of Bacillota; **Increase** of potential beneficial bacterial genera (*Thermomonas*,* Pseudomonas*,* Lactococcus*, and *Rhodanobacter*)	A, E	(Qiao et al. 2017)
*Bacillus amyloliquefaciens* GB03	**Antagonistic functions**: VOCs production; **Activating ISR**	Tomato	Pot experiment	14 days after treatment	NGS (Illumina MiSeq) − 16 S rRNA	**Increase** bacterial diversity; Microbiome shift (VOCs)	B, E	(Kong et al. 2021)
*Bacillus cereus* DW019	**Nutrient availability**: N_2_-fixing, siderophores production; **Plant hormone modulation**: IAA production	Cherry tomato	Pot experiment	70 days after inoculation	NGS − 16 S rRNA	**Increase** of Pseudomonadota and Actinomycetota; **Decrease** of Gemmatimonadota, Acidobacteriota, and Myxococcota	E	(Dong et al. 2023)
*Bacillus subtilis* NCD-2	**Antagonistic functions**: fengycin and surfactin production; **Activating ISR**	Tomato	Pot experiment	35 days after inoculation	NGS (Illumina MiSeq) − 16 S rRNA & ITS; qPCR - strain persistence	**Increase** of Pseudomonadota and Bacillota; **Increase** of potential beneficial bacterial genera (*Bacillus*,* Sphingobium*, and *Ramlibacter*); **Increase** of *Fusarium*,* Clonostachys*, and *Neurospora*; **Decrease** of *Byssochlamys*,* Aspergillus*, and *Fibulochlamys*	E	(Zhao et al. 2023)
*Bacillus amyloliquefaciens* CECT8238; SynCom consortium	NA	Tomato	Greenhouse experiment	At 8 week	NGS (Illumina MiSeq or NovaSeq6000) − 16 S rRNA & ITS	Different effects with single strains and SynCom; **Increase** of potential beneficial bacterial genera (*Blastococcus*, *Ramilibacter*, and *Ohtaekwangia*)	E	(Lee Díaz et al. 2024)
*Bacillus amyloliquefaciens* L-S60	Fungicidal properties	Cucumber	Greenhouse experiment	5, 10, 15, 20 and 25 days after sprout	NGS (Illumina HiSeq2500) − 16 S rRNA	**Increase** of potential beneficial bacterial genera (*Paenibacillus*, *Rhodanobacter*, and *Pseudomonas*) **Decrease** of Acidobacteriota and Gemmatimonadota	E	(Qin et al. 2017)
*Paenibacillus polymyxa* NSY50	Fungicidal properties	Cucumber	Pot experiment	30 days after inoculation with FOC	NGS (Illumina MiSeq) − 16 S rRNA & ITS	**Increase** of potential beneficial bacterial genera (*Bacillus*,* Actinobacteria*,* Streptomyces*,* Actinospica*,* Catenulispora*, and *Pseudomonas*); **Decrease** of potential pathogens (*Fusarium*)	D, E, F	(Shi et al. 2017)
*Bacillus amyloliquefaciens* FH-1	**Antagonistic functions**; **Nutrient availability**: N_2_-fixing, phosphate and potassium solubilization, siderophores production; **Plant hormone modulation**: ACC deaminase production	Cucumber	Pot experiment	35 days after sowing	NGS (Illumina MiSeq) − 16 S rRNA	**Increase** of Myxococcota, *Nannocystaceae*; **Decrease** of Acidobacteriota	E	(Wang et al. 2021)
*Bacillus subtilis* S1	Fungicidal properties	Cucumber	Pot experiment	15, 30 and 45 days after last treatment	NGS (Illumina MiSeq 300) − 16 S rRNA	Transient microbiome shift (on day 15 and 30): **Increase** of potential beneficial bacterial families (*Xanthomonadaceae*, *Comamonadaceae*, Cyclobactericae, *Rhodobacteraceae*, and *Acetobacteraceae*)	A	(Zhang et al. 2024)
*Bacillus velezensis* NJAU-Z9	**Nutrient availability**: ammonia production; **Plant hormone modulation**: IAA production	Pepper	Field experiment	At seedling, flowering and mature state	NGS (Illumina MiSeq) − 16 S rRNA & ITS; qPCR - strain persistence	**Increase** bacterial diversity, **decrease** fungal diversity; **Increase** of potential beneficial bacterial genera (*Sphingomonas*,* Sphingopyxis*,* Bradyrhizobium*,* Chitinophaga*,* Dyadobacter*,* Streptomyces*,* Lysobacter*,* Pseudomonas*, and *Rhizomicrobium*); **Increase** of potential beneficial fungal genera (*Cladorrhinum*,* Cladosporium*, and *Aspergillus*)	B, E	(Zhang et al. 2019)
*Bacillus subtilis* Ydj3	**Plant hormone modulation**: IAA production	Pepper	Field experiment	At the beginning of planting and at harvest	NGS (Illumina MiSeq 300) − 16 S rRNA Scanning electron microscope - root colonization assay	**Increase** of potential beneficial bacterial genera (*Nocardioides*, *Bacillus*, and *Gemmatimonas*)	E	(Liang et al. 2022)
*Bacillus subtilis* NCD-2	**Antagonistic functions**: fengycin and surfactin production; **Activating ISR**	Pepper	Pot experiment	35 days after inoculation	NGS (Illumina MiSeq) − 16 S rRNA & ITS; qPCR - strain persistence	**Increase** of Pseudomonadota and Bacillota; **Increase** of potential beneficial bacterial genera (*Bacillus*, *Sphingobium*, and *Lysobacter*); **Decrease** of Actinomycetota, Acidobacteriota, and Cyanobacteriota; **Increase** of *Byssochlamys*,* Acremonium*, and *Peziza*; **Decrease** of *Verticillium*,* Nectria*, and *Guehomyces*	E	(Zhao et al. 2023)
*Bacillus velezensis* FZB42	**Antagonistic functions**: fengycin, surfactin, bacillomycin D, macrolactin, bacilysin, difficidin and VOCs production **Plant hormone modulation**: IAA production **Activating ISR**	Lettuce	Pot and field experiment	At planting, 2 and 4 weeks after planting (pot); at planting, 2 and 5 weeks after planting (field)	T-RFLP	Transient microbiome shift	A	(Chowdhury et al. 2013)
*Bacillus velezensis* FZB42	**Antagonistic functions**: fengycin, surfactin, bacillomycin D, macrolactin, bacilysin, difficidin and VOCs production **Plant hormone modulation**: IAA production **Activating ISR**	Lettuce	Field experiment	At planting, 2 weeks and 5 weeks after planting	WGS	Transient microbiome shift	A	(Kröber et al. 2014)
*Bacillus velezensis* SAAS-63	NA	Lettuce	Pot experiment	30 days after the first treatment	NGS (Illumina MiSeq) − 16 S rRNA & ITS; UHPLC-MS/MS	**Increase** of Verrucomicrobiota and Chloroflexota under nutrient deficiency; **Increase** of beneficia bacterial taxa (*Streptomyces* and *Actinoallomurus)* under nutrient deficiency	E	(Bai et al. 2024)
*Bacillus amyloliquefaciens* G02	**Nutrient availability**: phosphate and selenium solubilization, siderophores production; **Plant hormone modulation**: IAA and ACC deaminase production	Lettuce	Pot experiment	Month after transplanting	NGS − 16 S rRNA	**Increase** of Bacillota, Acidobacteriota, and Cyanobacteriota; **Increase** of potential beneficial bacterial genera (*Bacillus*,* Vicinamibacterales*,* Sphingomonas*,* Intrasporangium*,* Roseiflexaceae*, and *Nocardioides*); **Decrease** of Chloroflexota, Myxococcota, and *Flavisolibacter*	E	(Huang et al. 2025)
*Brevibacillus laterosporus* AMCC100017	**Plant hormone modulation**: IAA and production	Potato	Pot experiment	Seedling, flowering, tuber bulking and harvesting stage	T-RFLP	**Increase** microbial diversity (in the flowering and the harvesting stage); Transient microbiome shift: **Increase** of potential beneficial bacterial taxa (*Bacillus*,* Pseudomonas*, and Actimomycetes)	A, B, E	(Chen et al. 2017)
*Brevibacillus laterosporus* BL12	Fungicidal properties	Potato	Pot experiment	Early tuber bulking, tuber bulking and harvesting stage	NGS (Illumina MiSeq PE300) − 16 S rRNA	**Increase** of Verrucomicrobiota; **Increase** of potential beneficial bacterial taxa (*Pseudomonas*,* Pseudarthrobacter*,* Brevibacillus*,* Massilia*,* Bosea*,* Devosia*, and *Peanibacillaceae*); **Decrease** of *Streptomyces*,* Parasegetibacter*,* Pedobacter*, and *Terrimonas*	D, E	(Li et al. 2021a)
*Bacillus subtilis* Bv17	NA	Potato	Field experiment	At two-month intervals, three times	NGS (Illumina HiSeq2500) − 16 S rRNA & ITS	**Increase** microbial diversity; **Increase** of Acidobacteriota; **Decrease** of Actinomycetota, *Alternaria* and *Humicola*	B, F	(Song et al. 2021)
*Bacillus amyloliquefaciens* QST713	Fungicidal properties	Potato	Field experiment	30 days after emergence	NGS (Illumina MiSeq) − 16 S rRNA & ITS	**Increase** of potential beneficial microbial genera (*Nitrosocosmicus* and *Sphingomonas*); **Decrease** of potential pathogens (*Fusarium*)	E, F	(Adamo et al. 2024)
*Bacillus subtilis* NCD-2	**Antagonistic functions**: fengycin and surfactin production; **Activating ISR**	Eggplant	Pot experiment	35 days after inoculation	NGS (Illumina MiSeq) − 16 S rRNA & ITS; qPCR - strain persistence	**Increase** of Pseudomonadota and Bacillota; **Increase** of potential beneficial bacterial genera (*Bacillus*,* Sphingobium*,* Lysobacter*, and *Ramlibacter*) **Increase** of *Byssochlamys*,* Penicillium*,* Mortierella*, and *Cladorrhinum*; **Decrease** of Actinomycetota, Acidobacteriota, and Cyanobacteriota; **Decrease** of *Fibulochlamys*	E	(Zhao et al. 2023)
*Bacillus subtilis* A-5	Fungicidal properties; γ-PGA production	Chinese cabbage	Pot experiment	60 days after planting	NGS (Illumina MiSeq PE300) − 16 S rRNA	**Increase** of Bacillota and *Micrococcaceae*; **Decrease** of Gemmatimonadota, Myxococcota, and Chloroflexota	E	(Bai et al. 2022)

### Orchard plants

The most commonly reported effects of Bacillota inoculation on the native microbiomes of orchard plants include shifts toward beneficial microbial groups and depletion of potential pathogens (Table 3).

Mahmoud et al. (2025) reported that *P. megaterium* B1L5 application to apple reduced early-stage root damage caused by apple replant disease and short-term decreased relative abundance of potential fungal pathogens: *Melanconiella* and *Fusarium*. Moreover, treatment increased the relative abundance of several genera known for their antifungal properties, such as *Luteimonas*, *Lysobacter*, *Pseudomonas*, *Sphingomonas*, *Paenibacillus*, *Flavobacterium*, *Rhodanobacter*, and *Pedobacter*. Corresponding pattern was reported in melon by Dong et al. (2025), where *P. polymyxa* NBmelon-1 also depleted *Melanconiella* and *Fusarium*, while increasing relative abundance of potentially beneficial taxa. A decrease in the relative abundance of potentially pathogenic taxa was also observed by Shen et al. (2015) in the banana rhizosphere after inoculation with *B. amyloliquefaciens* NJN-6. Furthermore, Tao et al. (2020) reported an increase in the indigenous *Pseudomonas* population after application of *B. amyloliquefaciens* W19. A similar pattern was noted by Shi et al. (2022a) in the rhizosphere of Chinese wild peach following inoculation with a *B. velezensis* consortium, which enriched several beneficial genera, including *Pseudomonas*, *Flavobacterium*, and *Arenimonas*. Consistently, Wang et al. (2022) demonstrated that application of *B. velezensis* SQR9 to pear rhizosphere increased the relative abundance of *Bacillus*, *Mycolicibacterium*, *Novosphingobium*, and *Sphingobium*, taxa associated with soil nutrient transformation. Shi et al. (2022b) examined the impact of three Bacillota inoculants, separately, on the American pecan rhizosphere microbiome. All of them decreased relative abundance of some potentially pathogenic fungal genera and increased relative abundance of several plant-growth promoting genera. However, all treatments also depleted some of the beneficial bacterial genera. Interestingly, all inoculants decreased relative abundance of *Pseudomonas*, *Pseudarthrobacter*, and *Ramlibacter*. Moreover, *Rhodovastum* was enriched by *Brevibacillus reuszeri* MPT17 application, but was depleted by *B. velezensis* YH20. Similarly, *Rhodanobacter* was enriched by *B. reuszeri* MPT17, while depleting by *B. pumilus* HR10 and *B. velezensis* YH20. Likewise, Xu et al. (2025) observed decrease in some potentially beneficial genera in peanut rhizosphere following inoculation with *B. laterosporus*. Treatment decreased relative abundance of *Sphingomonas* and *Bacillus*, increased bacterial diversity, and depleted *Fusarium*.

**Table 3:** Reported impact of Bacillota inoculation on the rhizosphere microbiota of orchard plants. Types of microbiome modulation described by Berg et al. (2021): (A) transient microbiome shifts, (B) stabilization or increase of microbial diversity, (C) stabilization or increase of microbiome evenness, (D) restoration of a dysbiosis/compensation or reduction of a pathogen-induced shift, (E) targeted shifts toward potential beneficial phyla, and (F) depletion of potential pathogens

Inoculant strain(s)	PGP traits	Plant	Type of experiments	Sampling	Impact detection method	Microbiome response	Microbiome modulation type	Reference
*Priestia megaterium* B1L5 (vegetative cells and spores)	Fungicidal properties	Apple	Pot experiment	6 and 33 days after inoculation	NGS − 16 S rRNA & ITS; qPCR - strain persistence (gfp gene); Confocal laser scanning microscope - gfp marked strain colonisation assay	**Increase** of potential beneficial bacterial genera (*Luteimonas*,* Lysobacter*,* Pseudomonas*,* Sphingomonas*,* Paenibacillus*,* Flavobacterium*,* Sphingobacterium*,* Rhodanobacter*, and *Pedobacter*); **Decrease** of potential pathogens (*Melanconiella* and *Fusarium*)	D, E, F	(Mahmoud et al. 2025)
*Paenibacillus polymyxa* NBmelon-1	Fungicidal properties	Melon	Greenhouse experiment	Autumn and spring (during fruit maturation)	NGS (Illumina MiSeq PE 250) − 16 S rRNA & ITS	**Increase** of Acidobacteriota and Bacillota; **Decrease** of potential pathogens (*Melanconiella* and *Fusarium*)	E, F	(Dong et al. 2025)
*Bacillus amyloliquefaciens* NJN-6	**Antagonistic functions**: iturin, bacillomycin D and macrolactin and VOCs production; **Plant hormone modulation**: IAA and GA3 production	Banana	Field experiment	At harvest (2 years after start of the experiment)	PCR-DGGE − 16 S rRNA & ITS	**Increase** microbial diversity; **Increase** of Acidobacteriota, Bacillota, Chloroflexota, Armatimonadota, Gemmatimonadota, and Nitrospirota; **Decrease** of Pseudomonadota and Ascomycota	B, E, F	(Shen et al. 2015)
*Bacillus amyloliquefaciens* W19	Fungicidal properties	Banana	Greenhouse experiment	After 4 months	NGS (Illumina NovaSeq 6000) − 16 S rRNA & ITS; qPCR (total bacteria, fungi, *Fusarium oxysporum*, *Bacillus* and *Pseudomonas*)	**Increase** of potential beneficial bacterial genera (*Bacillus* and *Pseudomonas*)	E	(Tao et al. 2020)
*Bacillus velezensis* YH-18 *and B. velezensis* YH-20	*Bacillus velezensis* YH-18: **Antagonistic functions**: antibacterial proteins, lipopeptides and VOCs production; **Nutrient availability**: N_2_-fixing *B. velezensis* YH-20: **Antagonistic functions**: antibacterial proteins, lipopeptides and VOCs production; **Nutrient availability**: increase available nitrogen and available potassium; **Plant hormone modulation**: IAA production	Chinese wild peach	Pot experiment	15 and 30 days after inoculation	NGS (Illumina MiSeq) − 16 S rRNA	**Increase** of potential beneficial bacterial genera (*Sphingomonas*, *Pseudomonas*,* Flavobacterium*,* Arenimonas*,* Aquicella*,* Devosia*,* Novosphingobium*, and *Ferruginibacter*); **Decrease** of *Bryobacter*,* Chryseolinea*,* Nitrospira*, and *Haliangium*	E	(Shi et al. 2022a)
*Bacillus velezensis* SQR9	**Antagonistic functions**: bacillibactin, bacillomycin D, fengycin, surfactin, bacillaene, difficidin, macrolactin, bacilysin and bacillunoic acid production; **Nutrient availability**: siderophores production; **Plant hormone modulation**: IAA production; **Activating ISR**	Pear	Field experiment	2 years after start of the experiment	WGS (Illumina NovaSeq 6000 S4)	**Increase** of potential beneficial bacterial genera (*Bacillus*,* Mycolicibacterium*,* Novosphingobium*, and *Sphingobium*)	E	(Wang et al. 2022)
*Bacillus pumilus* HR10	Fungicidal properties; **Activating ISR**	American pecan	Pot experiment	15 days after inoculation	NGS (Illumina NovaSeq 6000) − 16 S rRNA & ITS	**Increase** of Bacillota; **Increase** of potential beneficial bacterial genera (*Sphingomonas*, *Chujaibacter*, *Bacillus*,* Acidipila*,* Bryobacter*, and *Mizugakiibacter);* **Decrease** of *Rhodanobacter*, *Pseudomonas*,* Massilia*,* Lysobacter*,* Nitrospira*, *Pseudarthrobacter*, and *Ramlibacter;* **Increase** of mycorrhizal fungi; **Decrease** of potential pathogens (*Didimella*)	E, F	(Shi et al. 2022b)
*Bacillus velezensis* YH20	**Antagonistic functions**: antibacterial proteins, lipopeptides and VOCs production; **Nutrient availability**: increase available nitrogen and available potassium; **Plant hormone modulation**: IAA production	American pecan	Pot experiment	15 days after inoculation	NGS (Illumina NovaSeq 6000) − 16 S rRNA & ITS	**Increase** of Actinomycetota; **Increase** of potential beneficial bacterial genera (*Sphingomonas*, *Chujaibacter*, *Acidibacter*,* Pseudolabrys*,* Acidipila*,* Brukholderia*,* Acidothermus*,* Granulicella*,* Bradyrhizobium*,* Streptomyces*,* Bryobacter*,* Devosia*, and *Actinospica*); **Decrease** of *Rhodanobacter*, *Pseudomonas*,* Lysobacter*,* Nitrospira*, *Pseudarthrobacter*,* Rhodovastum*, and *Ramlibacter;* **Increase** of mycorrhizal fungi; **Decrease** of potential pathogens (*Didimella*)	E, F	(Shi et al. 2022b)
*Brevibacillus reuszeri* MPT17	**Nutrient availability**: increase available phosphorus and available potassium	American pecan	Pot experiment	15 days after inoculation	NGS (Illumina NovaSeq 6000) − 16 S rRNA & ITS	**Increase** of potential beneficial bacterial genera (*Rhodanobacter*, *Chujaibacter Gemmatimonas*,* Acidipila*,* Brukholderia*,* Reyranella*,* Rhodovastum*, and *Aquicella*); **Decrease** of *Pseudomonas*, *Massilia*,* Pseudarthrobacter*, and *Ramlibacter;* **Increase** of mycorrhizal fungi; **Decrease** of potential pathogens (*Fusarium* and *Didimella*)	E, F	(Shi et al. 2022b)
*Brevibacillus laterosporus*	Fungicidal properties; **Nutrient availability**: ligninolytic enzymes	Peanut	Pot experiment	120 days after planting	NGS (Illumina MiSeq) − 16 S rRNA & ITS	**Increase** bacterial diversity; **Increase** of *Anaerolinea* and *Anaeromyxobacter*; **Decrease** of *Gemmatimonas*,* Sphingomonas*, and *Bacillus*; **Decrease** of potential pathogens (*Fusarium*)	B, F	(Xu et al. 2025)

## Fiber plants

Research on the impact of Bacillota inoculation on the rhizosphere of fiber crops has focused predominantly on cotton, while other economically important species such as hemp or flax remain largely overlooked. In cotton, the most frequently reported type of microbial modulation is a shift in the rhizosphere microbiome toward beneficial microbial groups (Table 4). This pattern is consistent with findings observed in other crops.

Qin et al. (2021) observed that inoculation with *B. circulans* GN03 increased relative abundance of *Lysobacter* and *Herbaspirillum*. Likewise, Zhao et al. (2023) reported that *B. subtilis* NCD-2 also increased relative abundance of *Lysobacter* and other beneficial genera, including *Bdellovibrio*, *Pseudomonas*, *Peredibacter*, *Limnobacter*, and *Pedobacter*. Correspondingly, Yu et al. (2025) noted enrichment of *Lysobacter* and *Arthrobacter*, *Pseudarthrobacter*, and *Flavobacterium*, following treatment with *B. amyloliquefaciens*. Xu et al. (2023) similarly reported that *Bacillus paralicheniformis* RP01 increased relative abundance of beneficial bacterial genera, such as *Streptomyces*, *Bradyrhizobium*, and *Acinetobacter*.

**Table 4:** Reported impact of Bacillota inoculation on the rhizosphere microbiota of fiber plants. Types of microbiome modulation described by Berg et al. (2021): (A) transient microbiome shifts, (B) stabilization or increase of microbial diversity, (C) stabilization or increase of microbiome evenness, (D) restoration of a dysbiosis/compensation or reduction of a pathogen-induced shift, (E) targeted shifts toward potential beneficial phyla, and (F) depletion of potential pathogens

Inoculant strain(s)	PGP traits	Plant	Type of experiments	Sampling	Impact detection method	Microbiome response	Microbiome modulation type	Reference
*Bacillus circulans* GN03	**Plant hormone modulation**: IAA, GA, BR, SA and JA production; **Activating ISR**	Cotton	Pot experiment	30 days after inoculation	NGS (Illumina MiSeq) − 16 S rRNA	**Increase** of potential beneficial bacterial genera (*Lysobacter* and *Herbaspirillum*); **Decrease** of *Geobacter*, *Curvibacter*, and *Methylocysti*	A, E	(Qin et al. 2021)
*Bacillus paralicheniformis* RP01	**Plant hormone modulation**: IAA, BR, SA and JA production;	Cotton	Pot experiment	30 days after inoculation	NGS (Illumina MiSeq) − 16 S rRNA	**Increase** of potential beneficial bacterial genera (*Streptomyces*, *Bradyrhizobium*, *Gemmatirosa*,* Candidatus_Solibacter*, and *Acinetobacter*)	E	(Xu et al. 2023)
*Bacillus subtilis* NCD-2	**Antagonistic functions**: fengycin and surfactin production; **Activating ISR**	Cotton	Pot experiment	35 days after inoculation	NGS (Illumina MiSeq) − 16 S rRNA & ITS; qPCR - strain persistence	**Increase** of Bacteroidota and Gemmatimonadota; **Increase** of potential beneficial bacterial genera (*Lysobacter*,* Bdellovibrio*,* Pseudomonas*,* Peredibacter*,* Limnobacter*, and *Pedobacter*); **Decrease** of Patescibacteriota, Cyanobacteriota; **Increase** of *Acremonium* and *Guehomyces*; **Decrease** of *Byssochlamys*,* Neurospora*, and *Arthrobotrys*	E	(Zhao et al. 2023)
*Bacillus amyloliquefaciens*	NA	Cotton	Field experiment	At harvest (2 years after start of the experiment)	WGS (Illumina NovaSeq X Plus)	**Increase** of potential beneficial bacterial genera (*Arthrobacter*,* Pseudarthrobacter*,* Flavobacterium*, and *Lysobacter*); **Decrease** of Acidobacteriota and Chloroflexota; **Increase** of potential beneficial fungal genera (*Glomus*); **Decrease** of potential pathogens (*Fusarium*)	E, F	(Yu et al. 2025)

**Fig. 2:**
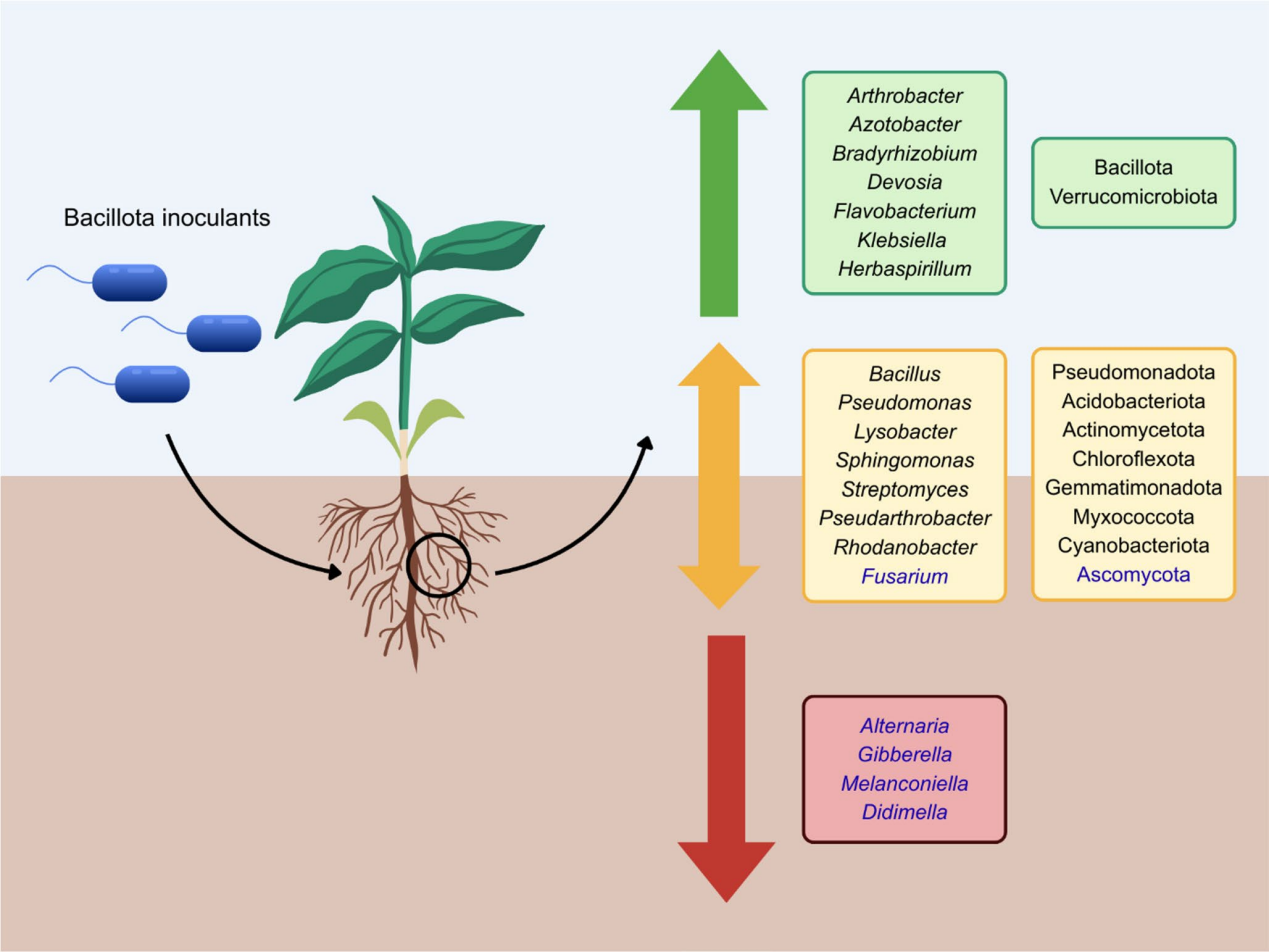
Schematic representation of the microbial taxa most frequently affected by Bacillota-based inoculants. Fungal taxa are marked in blue, and bacterial taxa in black. Taxa highlighted in green boxes have been reported exclusively as enriched, those in yellow boxes as either enriched or depleted, and those in red boxes exclusively as depleted

Studies on PGPR from the Bacillota phylum consistently demonstrate that their inoculation most commonly induces targeted shifts in the rhizosphere microbiome. Across cereals, vegetables, orchard crops, and fiber plants, the dominant pattern is a shift toward beneficial taxa and depletion of potential pathogens, particularly *Fusarium* (Fig. 2). The most frequently enriched bacterial genera include *Bacillus*, *Pseudomonas*, *Lysobacter*, *Sphingomonas*, *Streptomyces*, *Azotobacter*, *Arthrobacter*, *Pseudarthrobacter*, *Bradyrhizobium*, *Devosia*, *Flavobacterium*, *Klebsiella*, *Herbaspirillum*, and *Rhodanobacter*. These taxa are recognized as plant-beneficial and are linked to key functions, such as nitrogen fixation, nutrient transformation, phytohormone production, and biocontrol. However, effects are not universally positive for all these groups. Several studies have reported depletion of genera typically considered beneficial, including *Bacillus* (Xu et al. 2025), *Pseudomonas* (Shi et al. 2022b), *Lysobacter* (Shi et al. 2022b), *Sphingomonas* (Xu et al. 2025; Patyka et al. 2025), *Streptomyces* (Li et al. 2021a), *Pseudarthrobacter* (Shi et al. 2022b), and *Rhodanobacter* (Shi et al. 2022b). Notably, Shi et al. (2022b) demonstrated that different Bacillota strains applied to the same plant species produced contrasting effects on some genera, for example *Rhodanobacter* was enriched by *B. reuszeri* MPT17, and depleted by *B. pumilus* HR10 and *B. velezensis* YH20. At the same time, certain strains showed reproducible effects across multiple crops. Zhao et al. (2023) examined the effect of inoculation with *B. subtilis* NCD-2 to five different plants and reported consistent results. Likewise, treatment with *P. triticisoli* BJ-18 induced comparable shifts in both maize and winter wheat rhizosphere (Li et al. 2021b, c). Overall, Bacillota inoculation most commonly enriches PGPR-associated taxa, increases bacterial diversity, and suppresses fungal pathogens. However, microbiome responses remain strongly strain- and plant-dependent, and comparative studies evaluating multiple inoculants within the same plant species or the same inoculant across multiple plants are still limited.

## Detection methods

The detected impact of PGPR inoculation strongly depends on the sampling time, experimental design, and, more broadly, on the methodological approaches employed (Renoud et al. 2022; Kool et al. 2023; Lin et al. 2024). The investigation of how PGPR inoculation modulates the native rhizosphere microbiota has evolved substantially over the past decades, following advances in molecular biology and high-throughput technologies. Early studies relied primarily on culture-based techniques and low-resolution molecular fingerprinting, whereas recent studies are mainly based on next-generation sequencing (NGS) and multi-omics approaches to capture comprehensive community changes (Lagos et al. 2015; Garg et al. 2024; Rajguru et al. 2024).

### PCR-based methods

Initially, analyses were dominated by molecular fingerprinting techniques such as PCR-DGGE (denaturing gradient gel electrophoresis), T-RFLP (terminal restriction fragment length polymorphism), SSCP (single-stranded conformational polymorphism), and ARISA (automated ribosomal intergenic spacer analysis) (Chowdhury et al. 2013; Schmidt et al. 2014; Shen et al. 2015). These approaches rely on the separation of PCR-amplified molecular marker genes fragments, mainly the 16 S rRNA gene for bacteria and internal transcribed spacer (ITS) region for fungi, to generate community profiles. Fingerprinting methods provide early insights into microbial community shifts following PGPR inoculation, however their interpretative power is limited by low taxonomic resolution, reduced sensitivity to rare taxa, and lack of functional information (Mawarda et al. 2020). Currently, PCR-based approaches, primarily quantitative PCR (qPCR), are more commonly applied to assess functional modulation of resident microbial communities rather than microbiome taxonomic shifts. Frequently targeted genes include functional genes, such as *nifH* (nitrogen fixation), *acdS* (ACC deaminase activity), *phoD* (phosphorus solubilization), *amoA* (nitrification), and *nosZ* (denitrification) or marker genes (Li et al. 2021c; Wang et al. 2023). By comparing gene copy numbers between inoculated and non-inoculated treatments, qPCR enables detection of functional shifts within the native microbiome, even when overall community composition remains relatively stable. This approach is particularly effective in capturing enrichment or suppression of specific native microbial populations and serves as a valuable complement to sequencing-based methods (Jian et al. 2020). In addition to detecting changes in microbiome function, qPCR is mainly used to track the persistence of an inoculated PGPR strain in the rhizosphere using strain-specific primers (Couillerot et al. 2010; Zhao et al. 2023).

### Sequencing-based methods

With the development of next generation sequencing (NGS), sequencing-based approaches have become state-of-the-art techniques for characterizing rhizosphere microbial communities. NGS technologies are often classified by read length as second generation (100 to 800 bp) and third generation (over 10,000 bp) (Satam et al. 2023; Garg et al. 2024). The most popular platforms for the second generation are Illumina MiSeq and HiSeq, and Ion Torrent Personal Genome Machine (PGM) (Hu et al. 2021). Second generation sequencing (SGS) workflow consists of creating single-stranded DNA libraries by DNA fragmentation, adapters ligation and amplification using PCR, sequencing by synthesis, and data analysis (Mandlik et al. 2024). Unlike first generation sequencing, millions of DNA fragments are sequenced simultaneously (mass parallel sequencing), thus increasing efficiency of sequencing and reducing costs. However, SGS still has some difficulties such as errors during PCR or hardships with assembling complex genome fragments (Hu et al. 2021). The third-generation sequencing (TGS) overcame most of these limitations. These technologies enable generating long sequences (> 10 kb) directly from native DNA, without creating libraries. Currently, two main platforms are used in TGS, Pacific Biosciences Single Molecule, Real-Time (PacBio SMRT) and Oxford Nanopore Technologies (ONT) (Scarano et al. 2024).

Metagenomics analysis, based on sequencing methods, can be divided into two main approaches. The first one, metabarcoding or targeted metagenomics is primarily used for the identification of the microorganism community composition and its diversity. In this approach, extracted DNA is amplified during PCR with specific primers targeting genes of interest, most commonly marker or functional genes (Zhao et al. 2023; Oulkhir et al. 2025). Amplicon sequencing of the 16 S rRNA gene or ITS region provides significantly higher taxonomic resolution than fingerprinting techniques, thus enabling detailed analyses of diversity and community composition at genus level. Targeted metagenomics enables detecting changes in rare taxa that were previously undetectable using PCR-based profiling (Morgan et al. 2017; Changey et al. 2022). This method is fast and cost-effective, however, its ability to predict functional potential is limited, as it relies mainly on taxonomic and relative abundance data. To overcome these limitations, whole-genome sequencing (WGS, global metagenomics, shotgun metagenomics) is increasingly applied. Unlike amplicon-based methods, shotgun metagenomics captures the entire genetic content of microbial communities, allowing assessment of both, taxonomic composition and functional potential (Li et al. 2021b; Zhu et al. 2023; Chang et al. 2024; Yu et al. 2025). WGS enables identification of crucial metabolic pathways related to nutrient cycling, antimicrobial compounds synthesis or phytohormones production, thereby providing border insight in microbiome functioning.

Importantly, advances in bioinformatic reconstruction now allow the recovery of metagenome-assembled genomes (MAGs) from shotgun datasets. MAGs enable the reconstruction of near-complete or partial genomes of individual microbial populations directly from environmental samples (Setubal 2021; Mirete et al. 2025). This enables the association of observed taxonomic variation with the functional potential encoded within reconstructed genomes, facilitating the attribution of metabolic capacities and biosynthetic pathways to specific microbial lineages. Consequently, MAG-based analyses provide a more integrative understanding of how changes in community composition translate into functional alterations within the microbiome, offering a powerful tool to connect structure and function in complex soil ecosystems (Wang et al. 2025; Naziębło et al. 2025; Dobrzyński et al. 2026a).

### Multi-omics

Metagenomics is a key method to understand composition of microbial communities. However, application of post-genomic approaches, such as metatranscriptomics and metaproteomics can promote a deeper understanding of gene functions and protein expression, thus unraveling complex interaction between PGPR inoculants and the native rhizosphere microbiota (Mmotla et al. 2025). Multi-omics is a gaining-attention strategy that combines different omics methods (metagenomics, metatranscriptomics, metaproteomics, and metabolomics), thus linking overall genetic potential with the actual gene expression and protein synthesis (Sahoo et al. 2025). While metagenomics provides information about the whole genetic potential of microbiota, metatranscriptomics focus only on functional genes expressed by viable bacteria microbes. It is a particularly effective method to assess the impacts of PGPR inoculation on microbial community function, such as enhancing or inhibiting important metabolic pathways (Liu et al. 2025; Ai et al. 2025). Metaproteomics and metabolomics analyze the content of proteins or metabolites produced by the microbial community. These approaches provide even deeper insight into microbiome interactions and function and are an efficient way to study interactions between inoculants, microbiome and host plant (Bai et al. 2024; Francioli et al. 2025; Patyka et al. 2025).

### Limitations

Although the number of studies investigating the impact of PGPR on native rhizosphere microbiota has increased substantially in recent years, significant methodological and conceptual limitations still constrain our understanding of these complex interactions. While numerous reports describe shifts in microbial community composition following inoculation often indicating enrichment of beneficial taxa or depletion of pathogens – comprehensive or long-term studies remain scarce.

First, most studies investigating the effects of PGPR on rhizosphere microbial communities evaluate community structure at a single sampling point, most commonly at the end of the experimental period. Only a limited number of reports incorporate sampling at different stages of plant development, and even fewer monitor microbial dynamics over extended time frames (Chen et al. 2017; Zhu et al. 2023). Consequently, it remains uncertain whether the reported shifts in microbiome composition reflect transient disturbances, short-term enrichment of specific taxa, or more stable and sustained restructuring of the indigenous microbial community. Considering that rhizosphere interactions are inherently dynamic and are shaped by plant developmental stage, seasonal fluctuations, and environmental variability, single end-point assessments provide only a partial and potentially misleading representation of PGPR–microbiome interactions (Ajilogba et al. 2022). Therefore, long-term studies encompassing multiple plant growth stages and successive growing seasons are essential to accurately evaluate the stability and ecological significance of PGPR-induced changes in native microbial communities.

Second, most of the available studies have been conducted under controlled greenhouse or pot conditions, typically using a single soil type (Tables 1, 2, 3 and 4). Even field experiments are often restricted to one location. However, soil physicochemical properties, such as pH, texture, organic matter content, and nutrient availability – are key determinants of microbial community structure and function. The limited number of multi-site and multi-soil investigations therefore constrains the assessment of reproducibility and broader applicability of the reported findings. As a consequence, it remains unclear whether the observed microbiome shifts are strongly context-dependent or reflect more generalizable patterns. Comparative studies conducted across contrasting soil types and environmental conditions are thus essential to determine the robustness, consistency, and ecological relevance of PGPR-induced modulation of native microbial communities.

Third, only a limited number of studies quantitatively relate the abundance or persistence of introduced strains to changes observed in native rhizobacterial communities. Although next-generation sequencing approaches (e.g., 16 S rRNA amplicon sequencing or metagenomics) are widely used to characterize community composition, they are less frequently complemented by quantitative methods such as strain-specific qPCR, persistence assays, or direct assessment of rhizosphere colonization (Zhang et al. 2019; Win et al. 2020; Zhao et al. 2023; Ye et al. 2024; Mahmoud et al. 2025). Consequently, the relationship between inoculant population dynamics and shifts in indigenous microbial taxa remains insufficiently resolved.

### Concluding remarks

Accumulating evidence demonstrates that inoculation with PGPR from the Bacillota phylum can significantly modulate native rhizosphere microbiota across a wide range of agriculturally relevant crops. The most frequently observed outcomes include enrichment of plant-beneficial taxa, suppression of pathogens, and increases in microbial diversity or evenness. These microbiome shifts were often correlated with improved plant growth, stress tolerance, and yield performance. However, responses are not universally consistent, in some cases, taxa commonly regarded as beneficial are depleted. Microbiome modulation is strongly strain- and plant-dependent, and influenced by environmental conditions, sampling terms and methodological approaches.

Despite major advances in sequencing technologies and multi-omics approaches, important knowledge gaps remain. Most studies rely on short-term experiments and single sampling points, limiting our ability to distinguish transient shifts from stable, ecologically meaningful restructuring of microbial communities. Furthermore, the persistence and colonization dynamics of introduced strains are rarely quantitatively linked to observed shifts in indigenous microbiota.

Future research should prioritize long-term, replicated experiments across multiple soil types and environmental conditions, combined with integrated structural and functional analyses. Linking multi-omics data with quantitative tracking of inoculant populations will be essential to establish causal relationships between PGPR application, microbiome modulation, and plant performance. Comparative studies evaluating multiple inoculants within the same crop, as well as single strains across diverse hosts, are also needed to disentangle strain-specific from host-driven effects.

Deeper understanding of PGPR-microbiome interactions is crucial for optimizing bioinoculant formulations, improving their persistence and consistency under field conditions, and ensuring ecological safety. Such knowledge will ultimately support the development of microbial-based strategies for sustainable and climate-resilient agriculture.

## Data Availability

No datasets were generated or analysed during the current study.

## References

[CR130] (2021) The state of the world’s land and water resources for food and agriculture – systems at breaking point (SOLAW 2021). FAO

[CR1] Adamo I, Acin-Albiac M, Röttjers S et al (2024) Short impact on soil microbiome of a *Bacillus amyloliquefaciens* QST713 based product that correlates with higher potato yield across USA. Front Plant Sci 15:1332840. 10.3389/fpls.2024.1332840 38545390 10.3389/fpls.2024.1332840 PMC10967024

[CR2] Ahmad Ansari F, Ahmad I, Pichtel J (2023) Synergistic effects of biofilm-producing PGPR strains on wheat plant colonization, growth and soil resilience under drought stress. Saudi J Biol Sci 30:103664. 10.1016/j.sjbs.2023.103664 37213696 10.1016/j.sjbs.2023.103664 PMC10193011

[CR3] Ai W, Qiu Y, Hua J et al (2025) *Pseudomonas fluorescens* P34 colonization impacts expression changes in wheat roots, reshapes rhizosphere microbial communities and promotes wheat plant growth. Microbiol Res 301:128306. 10.1016/j.micres.2025.128306 40782586 10.1016/j.micres.2025.128306

[CR4] Ajilogba CF, Olanrewaju OS, Babalola OO (2022) Plant Growth Stage Drives the Temporal and Spatial Dynamics of the Bacterial Microbiome in the Rhizosphere of *Vigna subterranea*. Front Microbiol 13:825377. 10.3389/fmicb.2022.825377 35250941 10.3389/fmicb.2022.825377 PMC8891599

[CR5] Ali Q, Khan AR, Yujie W et al (2025) Antimicrobial metabolites of *Bacillus velezensis* FZB42 reshape rice rhizosphere microbial community composition and induce host resistance against *Rhizoctonia solani*. Curr Plant Biol 41:100440. 10.1016/j.cpb.2025.100440

[CR6] Bai N, Zhang H, He Y et al (2022) Effects of *Bacillus subtilis* A-5 and its fermented γ-polyglutamic acid on the rhizosphere bacterial community of Chinese cabbage. Front Microbiol 13:954489. 10.3389/fmicb.2022.954489 36046026 10.3389/fmicb.2022.954489 PMC9421268

[CR7] Bai Y, Song K, Gao M et al (2024) Using multi-omics to explore the effect of *Bacillus velezensis* SAAS-63 on resisting nutrient stress in lettuce. Appl Microbiol Biotechnol 108:313. 10.1007/s00253-024-13153-y 38683244 10.1007/s00253-024-13153-y PMC11058974

[CR8] Bakker PAHM, Berendsen RL, Doornbos RF et al (2013) The rhizosphere revisited: root microbiomics. Front Plant Sci 4:165. 10.3389/fpls.2013.00165 23755059 10.3389/fpls.2013.00165 PMC3667247

[CR9] Berg G, Kusstatscher P, Abdelfattah A et al (2021) Microbiome Modulation—Toward a Better Understanding of Plant Microbiome Response to Microbial Inoculants. Front Microbiol 12:650610. 10.3389/fmicb.2021.650610 33897663 10.3389/fmicb.2021.650610 PMC8060476

[CR10] Besset-Manzoni Y, Rieusset L, Joly P et al (2018) Exploiting rhizosphere microbial cooperation for developing sustainable agriculture strategies. Environ Sci Pollut Res 25:29953–29970. 10.1007/s11356-017-1152-2 10.1007/s11356-017-1152-2 29313197

[CR11] Bouchard-Rochette M, Machrafi Y, Cossus L et al (2022) *Bacillus pumilus* PTB180 and *Bacillus subtilis* PTB185: Production of lipopeptides, antifungal activity, and biocontrol ability against *Botrytis cinerea*. Biol Control 170:104925. 10.1016/j.biocontrol.2022.104925

[CR13] Changey F, Nunan N, Herrmann AM, Lerch TZ (2022) Catching change in microbial diversity indicators under different soil organic matter managements: Higher taxonomic resolution, better discrimination? Ecol Indic 139:108897. 10.1016/j.ecolind.2022.108897

[CR12] Chang W, Chen W, Hu Y, Wang Z (2024) *Bacillus altitudinis* LZP02 improves rice growth by reshaping the rhizosphere microbiome. Plant Soil 498:279–294. 10.1007/s11104-023-06435-3

[CR14] Chebotar VK, Chizhevskaya EP, Andronov EE et al (2023) Assessment of the Rhizosphere Bacterial Community under Maize Growth Using Various Agricultural Technologies with Biomodified Mineral Fertilizers. Agronomy 13:1855. 10.3390/agronomy13071855

[CR15] Chen Q, Song Y, An Y et al (2024) Mechanisms and Impact of Rhizosphere Microbial Metabolites on Crop Health, Traits, Functional Components: A Comprehensive Review. Molecules 29:5922. 10.3390/molecules29245922 39770010 10.3390/molecules29245922 PMC11679325

[CR16] Chen S, Zhang M, Wang J et al (2017) Biocontrol effects of *Brevibacillus laterosporus* AMCC100017 on potato common scab and its impact on rhizosphere bacterial communities. Biol Control 106:89–98. 10.1016/j.biocontrol.2017.01.005

[CR17] Chepsergon J, Moleleki LN (2023) Rhizosphere bacterial interactions and impact on plant health. Curr Opin Microbiol 73:102297. 10.1016/j.mib.2023.102297 37002974 10.1016/j.mib.2023.102297

[CR18] Chowdhury SP, Dietel K, Rändler M et al (2013) Effects of *Bacillus amyloliquefaciens* FZB42 on Lettuce Growth and Health under Pathogen Pressure and Its Impact on the Rhizosphere Bacterial Community. PLoS ONE 8:e68818. 10.1371/journal.pone.0068818 23935892 10.1371/journal.pone.0068818 PMC3720850

[CR19] Cotta SR, Dias ACF, Mendes R, Andreote FD (2025) Role of horizontal gene transfer and cooperation in rhizosphere microbiome assembly. Braz J Microbiol 56:225–236. 10.1007/s42770-024-01583-9 39730778 10.1007/s42770-024-01583-9 PMC11885732

[CR20] Couillerot O, Poirier M-A, Prigent‐Combaret C et al (2010) Assessment of SCAR markers to design real‐time PCR primers for rhizosphere quantification of *Azospirillum brasilense* phytostimulatory inoculants of maize. J Appl Microbiol 109:528–538. 10.1111/j.1365-2672.2010.04673.x 20141548 10.1111/j.1365-2672.2010.04673.x

[CR21] De Andrade LA, Santos CHB, Frezarin ET et al (2023) Plant Growth-Promoting Rhizobacteria for Sustainable Agricultural Production. Microorganisms 11:1088. 10.3390/microorganisms11041088 37110511 10.3390/microorganisms11041088 PMC10146397

[CR22] Dobrzyński J, Gradowski M, Radkowski A, Bujak H (2026a) Chloroflexota in agricultural soils: current knowledge and future research directions. Front Microbiol 17:1705889. 10.3389/fmicb.2026.1705889 41695957 10.3389/fmicb.2026.1705889 PMC12901349

[CR23] Dobrzyński J, Jakubowska Z (2025) *Pseudomonas protegens* as a biocontrol agent against phytopathogenic fungi - mini review. World J Microbiol Biotechnol 41:428. 10.1007/s11274-025-04643-w 41174125 10.1007/s11274-025-04643-w PMC12578757

[CR24] Dobrzyński J, Jakubowska Z, Kulkova I et al (2023) Biocontrol of fungal phytopathogens by *Bacillus pumilus*. Front Microbiol 14:1194606. 10.3389/fmicb.2023.1194606 37560520 10.3389/fmicb.2023.1194606 PMC10407110

[CR25] Dobrzyński J, Kulkova I, Jakubowska Z et al (2026b) *Raoultella* sp. KUP47 Promotes *Dactylis Glomerata* L. Growth and Enriches Verrucomicrobiota in the Rhizosphere. J Soil Sci Plant Nutr. 10.1007/s42729-026-03133-8

[CR26] Dobrzyński J, Naziębło A, Kulkova I et al (2026c) Response of triticale and its native rhizobacterial community to inoculation with a consortium of *Paenibacillus* sp. Z15 and *Pseudomonas* sp. KR227. World J Microbiol Biotechnol 42:91. 10.1007/s11274-026-04851-y 41706256 10.1007/s11274-026-04851-y

[CR27] Dobrzyński J, Naziębło A, Kulkova I et al (2026d) *Paenibacillus*–*Pseudomonas* Consortium Improves Barley Performance with Minimal Impact on Native Rhizobacterial Community. Microorganisms 14:488. 10.3390/microorganisms14020488 41753774 10.3390/microorganisms14020488 PMC12943435

[CR28] Dong W, Liu H, Ning Z et al (2023) Inoculation with *Bacillus cereus* DW019 Modulates Growth, Yield and Rhizospheric Microbial Community of Cherry Tomato. Agronomy 13:1458. 10.3390/agronomy13061458

[CR29] Dong W, Zang Q, Wang Y et al (2025) Synergistic Effects of *Paenibacillus polymyxa* NBmelon-1 Inoculation and Grafting Restructure of Rhizosphere Microbiome and Enhanced Disease Resistance in Melon Self-Rootstocks. Microorganisms 13:1172. 10.3390/microorganisms13061172 40572060 10.3390/microorganisms13061172 PMC12194880

[CR30] Drewnowska JM, Fiodor A, Barboza-Corona JE, Swiecicka I (2020) Chitinolytic activity of phylogenetically diverse *Bacillus cereus* sensu lato from natural environments. Syst Appl Microbiol 43:126075. 10.1016/j.syapm.2020.126075 32173136 10.1016/j.syapm.2020.126075

[CR31] Fatouros G, Gkizi D, Fragkogeorgi GA et al (2018) Biological control of *Pythium*, *Rhizoctonia* and *Sclerotinia* in lettuce: association of the plant protective activity of the bacterium *Paenibacillus alvei* K165 with the induction of systemic resistance. Plant Pathol 67:418–425. 10.1111/ppa.12747

[CR32] Francioli D, Kampouris ID, Kuhl-Nagel T et al (2025) Microbial inoculants modulate the rhizosphere microbiome, alleviate plant stress responses, and enhance maize growth at field scale. Genome Biol 26:148. 10.1186/s13059-025-03621-7 40452057 10.1186/s13059-025-03621-7 PMC12128319

[CR33] Fujita S, Yokota K (2019) Disease suppression by the cyclic lipopeptides iturin A and surfactin from *Bacillus* spp. against *Fusarium* wilt of lettuce. J Gen Plant Pathol 85:44–48. 10.1007/s10327-018-0816-1

[CR34] Garbeva P, Hordijk C, Gerards S, De Boer W (2014) Volatile-mediated interactions between phylogenetically different soil bacteria. Front Microbiol 5:289. 10.3389/fmicb.2014.00289 24966854 10.3389/fmicb.2014.00289 PMC4052926

[CR35] Garg D, Patel N, Rawat A, Rosado AS (2024) Cutting edge tools in the field of soil microbiology. Curr Res Microb Sci 6:100226. 10.1016/j.crmicr.2024.100226 38425506 10.1016/j.crmicr.2024.100226 PMC10904168

[CR36] Goud MS, Sharma SK, Kharbikar LL et al (2025) *Bacillus* species consortium with tryptophan-dependent and -independent pathways mediated production of IAA and its derivatives modulates soil biological properties, growth and yield of wheat. Plant Soil 508:71–97. 10.1007/s11104-024-06782-9

[CR37] Gu Y, Yan W, Chen Y et al (2025) Plant growth-promotion triggered by extracellular polymer is associated with facilitation of bacterial cross-feeding networks of the rhizosphere. ISME J Wraf 040. 10.1093/ismejo/wraf040 10.1093/ismejo/wraf040 PMC11937826 40037574

[CR38] Hossain A, Ali MA, Lin L et al (2023) Biocontrol of Soft Rot Dickeya and *Pectobacterium* Pathogens by Broad-Spectrum Antagonistic Bacteria within *Paenibacillus polymyxa* Complex. Microorganisms 11:817. 10.3390/microorganisms11040817 37110240 10.3390/microorganisms11040817 PMC10142376

[CR40] Huang B, Li B, Pan G et al (2025) *Bacillus amyloliquefaciens* G02 enhances selenium uptake in lettuce (*Lactuca sativa*) by improving soil selenium availability and rhizosphere microbiome regulation. Front Microbiol 16:1656037. 10.3389/fmicb.2025.1656037 41078508 10.3389/fmicb.2025.1656037 PMC12512348

[CR41] Hussen YA, Geleta K, Alemu M (2026) The economic impact of climate change on agriculture: a meta-analysis. Agric Food Secur 15:13. 10.1186/s40066-025-00596-3

[CR39] Hu T, Chitnis N, Monos D, Dinh A (2021) Next-generation sequencing technologies: An overview. Hum Immunol 82:801–811. 10.1016/j.humimm.2021.02.012 33745759 10.1016/j.humimm.2021.02.012

[CR42] Jian C, Luukkonen P, Yki-Järvinen H et al (2020) Quantitative PCR provides a simple and accessible method for quantitative microbiota profiling. PLoS ONE 15:e0227285. 10.1371/journal.pone.0227285 31940382 10.1371/journal.pone.0227285 PMC6961887

[CR43] Jiang J, Gao L, Bie X et al (2016) Identification of novel surfactin derivatives from NRPS modification of *Bacillus subtilis* and its antifungal activity against *Fusarium moniliforme*. BMC Microbiol 16:31. 10.1186/s12866-016-0645-3 26957318 10.1186/s12866-016-0645-3 PMC4784341

[CR44] Kazerooni EA, Maharachchikumbura SSN, Adhikari A et al (2021) Rhizospheric *Bacillus amyloliquefaciens* Protects *Capsicum annuum* cv. Geumsugangsan From Multiple Abiotic Stresses via Multifarious Plant Growth-Promoting Attributes. Front Plant Sci 12:669693. 10.3389/fpls.2021.669693 34113368 10.3389/fpls.2021.669693 PMC8185346

[CR45] Khoso MA, Wagan S, Alam I et al (2024) Impact of plant growth-promoting rhizobacteria (PGPR) on plant nutrition and root characteristics: Current perspective. Plant Stress 11:100341. 10.1016/j.stress.2023.100341

[CR46] Kong HG, Song GC, Sim H-J, Ryu C-M (2021) Achieving similar root microbiota composition in neighbouring plants through airborne signalling. ISME J 15:397–408. 10.1038/s41396-020-00759-z 32973341 10.1038/s41396-020-00759-z PMC8027813

[CR47] Kool J, Tymchenko L, Shetty SA, Fuentes S (2023) Reducing bias in microbiome research: Comparing methods from sample collection to sequencing. Front Microbiol 14:1094800. 10.3389/fmicb.2023.1094800 37065158 10.3389/fmicb.2023.1094800 PMC10101209

[CR48] Kröber M, Wibberg D, Grosch R et al (2014) Effect of the strain *Bacillus amyloliquefaciens* FZB42 on the microbial community in the rhizosphere of lettuce under field conditions analyzed by whole metagenome sequencing. Front Microbiol 5:252. 10.3389/fmicb.2014.00252 24904564 10.3389/fmicb.2014.00252 PMC4033844

[CR49] Lagos L, Maruyama F, Nannipieri P et al (2015) Current overview on the study of bacteria in the rhizosphere by modern molecular techniques: a mini review. J Soil Sci Plant Nutr 15(2):504–523. 10.4067/S0718-95162015005000042

[CR50] Laishram B, Devi OR, Dutta R et al (2025) Plant-microbe interactions: PGPM as microbial inoculants/biofertilizers for sustaining crop productivity and soil fertility. Curr Res Microb Sci 8:100333. 10.1016/j.crmicr.2024.100333 39835267 10.1016/j.crmicr.2024.100333 PMC11743900

[CR51] Leach JE, Triplett LR, Argueso CT, Trivedi P (2017) Communication in the Phytobiome. Cell 169:587–596. 10.1016/j.cell.2017.04.025 28475891 10.1016/j.cell.2017.04.025

[CR52] Lee Díaz AS, Minchev Z, Raaijmakers JM et al (2024) Impact of bacterial and fungal inoculants on the resident rhizosphere microbiome and the volatilome of tomato plants under leaf herbivory stress. FEMS Microbiol Ecol 100:fiad160. 10.1093/femsec/fiad160 38331428 10.1093/femsec/fiad160 PMC10858387

[CR57] Liang Y-R, Liao F-C, Huang T-P (2022) Deciphering the influence of *Bacillus subtilis* strain Ydj3 colonization on the vitamin C contents and rhizosphere microbiomes of sweet peppers. PLoS ONE 17:e0264276. 10.1371/journal.pone.0264276 35226695 10.1371/journal.pone.0264276 PMC8884494

[CR53] Li C, Shi W, Wu D et al (2021a) Biocontrol of potato common scab by *Brevibacillus laterosporus* BL12 is related to the reduction of pathogen and changes in soil bacterial community. Biol Control 153:104496. 10.1016/j.biocontrol.2020.104496

[CR54] Li J, Wang J, Liu H et al (2022) Application of microbial inoculants significantly enhances crop productivity: A meta-analysis of studies from 2010 to 2020. J Sustain Agric Environ 1:216–225. 10.1002/sae2.12028

[CR58] Lin H-H, Torres M, Adams CA et al (2024) Impact of Inoculation Practices on Microbiota Assembly and Community Stability in a Fabricated Ecosystem. Phytobiomes J 8:155–167. 10.1094/PBIOMES-06-23-0050-R

[CR59] Liu H, Xiong Y, Li D et al (2025) Impact of pest-induced *Burkholderia* inoculation on tea seedings: Integrated rhizosphere microbiome, transcriptome and metabolome profiling. Ind Crops Prod 232:121341. 10.1016/j.indcrop.2025.121341

[CR60] Liu W, Mu W, Zhu B et al (2008) Antagonistic Activities of Volatiles from Four Strains of *Bacillus* spp. and *Paenibacillus* spp. Against Soil-Borne Plant Pathogens. Agric Sci China 7:1104–1114. 10.1016/S1671-2927(08)60153-4

[CR55] Li Y, Li Q, Chen S (2021b) Diazotroph *Paenibacillus triticisoli* BJ-18 Drives the Variation in Bacterial, Diazotrophic and Fungal Communities in the Rhizosphere and Root/Shoot Endosphere of Maize. Int J Mol Sci 22:1460. 10.3390/ijms22031460 33540521 10.3390/ijms22031460 PMC7867140

[CR56] Li Y, Wang M, Chen S (2021c) Application of N2-fixing *Paenibacillus triticisoli* BJ-18 changes the compositions and functions of the bacterial, diazotrophic, and fungal microbiomes in the rhizosphere and root/shoot endosphere of wheat under field conditions. Biol Fertil Soils 57:347–362. 10.1007/s00374-020-01528-y

[CR61] Lopes MJDS, Dias-Filho MB, Gurgel ESC (2021) Successful Plant Growth-Promoting Microbes: Inoculation Methods and Abiotic Factors. Front Sustain Food Syst 5:606454. 10.3389/fsufs.2021.606454

[CR62] Mahapatra S, Yadav R, Ramakrishna W (2022) *Bacillus subtilis* impact on plant growth, soil health and environment: Dr. Jekyll and Mr. Hyde. J Appl Microbiol 132:3543–3562. 10.1111/jam.15480 35137494 10.1111/jam.15480

[CR63] Mahmoud FM, Edelmann H, Si Y et al (2025) Transient colonization by *Priestia megaterium* B1L5 alters the structure of the rhizosphere microbiome towards potential plant beneficial bacterial groups in apple plantlets. Environ Microbiome 20:104. 10.1186/s40793-025-00762-x 40804700 10.1186/s40793-025-00762-x PMC12344982

[CR64] Mandlik JS, Patil AS, Singh S (2024) Next-Generation Sequencing (NGS): Platforms and Applications. J Pharm Bioallied Sci 16:S41–S45. 10.4103/jpbs.jpbs_838_23 38595613 10.4103/jpbs.jpbs_838_23 PMC11001031

[CR65] Mawarda PC, Le Roux X, Van Dirk J, Salles JF (2020) Deliberate introduction of invisible invaders: A critical appraisal of the impact of microbial inoculants on soil microbial communities. Soil Biol Biochem 148:107874. 10.1016/j.soilbio.2020.107874

[CR66] Mirete S, Sánchez-Costa M, Díaz-Rullo J et al (2025) Metagenome-Assembled Genomes (MAGs): Advances, Challenges, and Ecological Insights. Microorganisms 13:985. 10.3390/microorganisms13050985 40431158 10.3390/microorganisms13050985 PMC12114606

[CR67] Mmotla K, Sibanyoni NR, Allie F et al (2025) Exploring the intricacies of plant growth promoting rhizobacteria interactions: an omics review. Ann Microbiol 75:5. 10.1186/s13213-025-01793-y

[CR68] Moore JAM, Abraham PE, Michener JK et al (2022) Ecosystem consequences of introducing plant growth promoting rhizobacteria to managed systems and potential legacy effects. New Phytol 234:1914–1918. 10.1111/nph.18010 35098533 10.1111/nph.18010 PMC9314638

[CR69] Morgan HH, Du Toit M, Setati ME (2017) The Grapevine and Wine Microbiome: Insights from High-Throughput Amplicon Sequencing. Front Microbiol 8:820. 10.3389/fmicb.2017.00820 28553266 10.3389/fmicb.2017.00820 PMC5425579

[CR70] Naziębło A, Pytlak A, Furtak A, Dobrzyński J (2025) Advances and Hotspots in Research on Verrucomicrobiota: Focus on Agroecosystems. Microb Ecol 89:1. 10.1007/s00248-025-02657-3 41275050 10.1007/s00248-025-02657-3 PMC12714770

[CR71] Ocwa A, Harsanyi E, Széles A et al (2023) A bibliographic review of climate change and fertilization as the main drivers of maize yield: implications for food security. Agric Food Secur 12:14. 10.1186/s40066-023-00419-3

[CR72] Orozco-Mosqueda MDC, Rocha-Granados MDC, Glick BR, Santoyo G (2018) Microbiome engineering to improve biocontrol and plant growth-promoting mechanisms. Microbiol Res 208:25–31. 10.1016/j.micres.2018.01.005 29551209 10.1016/j.micres.2018.01.005

[CR73] Oulkhir FE, Allaoui A, Idbella A et al (2025) *Bacillus subtilis* ED24 Controls *Fusarium culmorum* in Wheat Through Bioactive Metabolite Secretion and Modulation of Rhizosphere Microbiome. Microb Ecol 88:89. 10.1007/s00248-025-02590-5 40830705 10.1007/s00248-025-02590-5 PMC12364970

[CR74] Pathan SI, Ceccherini MT, Sunseri F, Lupini A (2020) Rhizosphere as Hotspot for Plant-Soil-Microbe Interaction. In: Datta R, Meena RS, Pathan SI, Ceccherini MT (eds) Carbon and Nitrogen Cycling in Soil. Springer Singapore, Singapore, pp 17–43

[CR75] Patyka M, Wang R, Honchar A et al (2025) Modulation of the rhizosphere microbiome structure and optimization of beneficial functions in winter wheat induced by *Bacillus subtilis*: a metagenomic and phenotypic study. FEMS Microbiol Ecol 101:fiaf097. 10.1093/femsec/fiaf097 41026097 10.1093/femsec/fiaf097 PMC12596712

[CR76] Pereira P, Inacio M, Barcelo D, Zhao W (2025) Impacts of agriculture intensification on biodiversity loss, climate change and ecosystem services. Curr Opin Environ Sci Health 46:100637. 10.1016/j.coesh.2025.100637

[CR77] Poria V, Dębiec-Andrzejewska K, Fiodor A et al (2022) Plant Growth-Promoting Bacteria (PGPB) integrated phytotechnology: A sustainable approach for remediation of marginal lands. Front Plant Sci 13:999866. 10.3389/fpls.2022.999866 36340355 10.3389/fpls.2022.999866 PMC9634634

[CR78] Poulaki EG, Tjamos SE (2023) *Bacillus* species: factories of plant protective volatile organic compounds. J Appl Microbiol 134:lxad037. 10.1093/jambio/lxad037 36822621 10.1093/jambio/lxad037

[CR79] Poveda J (2021) Beneficial effects of microbial volatile organic compounds (MVOCs) in plants. Appl Soil Ecol 168:104118. 10.1016/j.apsoil.2021.104118

[CR80] Qiao J, Yu X, Liang X et al (2017) Addition of plant-growth-promoting *Bacillus subtilis* PTS-394 on tomato rhizosphere has no durable impact on composition of root microbiome. BMC Microbiol 17:131. 10.1186/s12866-017-1039-x 28583081 10.1186/s12866-017-1039-x PMC5460418

[CR81] Qin L, Tian P, Cui Q et al (2021) *Bacillus circulans* GN03 Alters the Microbiota, Promotes Cotton Seedling Growth and Disease Resistance, and Increases the Expression of Phytohormone Synthesis and Disease Resistance-Related Genes. Front Plant Sci 12:644597. 10.3389/fpls.2021.644597 33936131 10.3389/fpls.2021.644597 PMC8079787

[CR82] Qin Y, Shang Q, Zhang Y et al (2017) *Bacillus amyloliquefaciens* L-S60 Reforms the Rhizosphere Bacterial Community and Improves Growth Conditions in Cucumber Plug Seedling. Front Microbiol 8:2620. 10.3389/fmicb.2017.02620 29312278 10.3389/fmicb.2017.02620 PMC5744474

[CR83] Radhakrishnan R, Hashem A, Abd_Allah EF (2017) *Bacillus*: A Biological Tool for Crop Improvement through Bio-Molecular Changes in Adverse Environments. Front Physiol 8:667. 10.3389/fphys.2017.00667 28932199 10.3389/fphys.2017.00667 PMC5592640

[CR84] Rafique M, Naveed M, Mumtaz MZ et al (2024) Unlocking the potential of biofilm-forming plant growth-promoting rhizobacteria for growth and yield enhancement in wheat (*Triticum aestivum* L). Sci Rep 14:15546. 10.1038/s41598-024-66562-4 38969785 10.1038/s41598-024-66562-4 PMC11226629

[CR85] Rajguru B, Shri M, Bhatt VD (2024) Exploring microbial diversity in the rhizosphere: a comprehensive review of metagenomic approaches and their applications. 3 Biotech 14:224. 10.1007/s13205-024-04065-9 39247454 10.1007/s13205-024-04065-9 PMC11379838

[CR86] Raza W, Yuan J, Ling N et al (2015) Production of volatile organic compounds by an antagonistic strain *Paenibacillus polymyxa* WR-2 in the presence of root exudates and organic fertilizer and their antifungal activity against *Fusarium oxysporum* f. sp. *niveum*. Biol Control 80:89–95. 10.1016/j.biocontrol.2014.09.004

[CR87] Renoud S, Abrouk D, Prigent-Combaret C et al (2022) Effect of Inoculation Level on the Impact of the PGPR *Azospirillum lipoferum* CRT1 on Selected Microbial Functional Groups in the Rhizosphere of Field Maize. Microorganisms 10:325. 10.3390/microorganisms10020325 35208780 10.3390/microorganisms10020325 PMC8877547

[CR88] Sahoo A, Yadav G, Mehta T et al (2025) Omics-driven insights into plant growth-promoting microorganisms for sustainable agriculture. Discov Sustain 6:659. 10.1007/s43621-025-01582-2

[CR89] Sasse J, Martinoia E, Northen T (2018) Feed Your Friends: Do Plant Exudates Shape the Root Microbiome? Trends Plant Sci 23:25–41. 10.1016/j.tplants.2017.09.003 29050989 10.1016/j.tplants.2017.09.003

[CR90] Satam H, Joshi K, Mangrolia U et al (2023) Next-Generation Sequencing Technology: Current Trends and Advancements. Biology 12:997. 10.3390/biology12070997 37508427 10.3390/biology12070997 PMC10376292

[CR91] Scarano C, Veneruso I, De Simone RR et al (2024) The Third-Generation Sequencing Challenge: Novel Insights for the Omic Sciences. Biomolecules 14:568. 10.3390/biom14050568 38785975 10.3390/biom14050568 PMC11117673

[CR92] Schmidt R, Köberl M, Mostafa A et al (2014) Effects of bacterial inoculants on the indigenous microbiome and secondary metabolites of chamomile plants. Front Microbiol 5:64. 10.3389/fmicb.2014.00064 24600444 10.3389/fmicb.2014.00064 PMC3928675

[CR93] Selim S, Negrel J, Wendehenne D et al (2010) Stimulation of Defense Reactions in *Medicago truncatula* by Antagonistic Lipopeptides from *Paenibacillus* sp. Strain B2. Appl Environ Microbiol 76:7420–7428. 10.1128/AEM.00171-10 20870792 10.1128/AEM.00171-10 PMC2976201

[CR94] Setubal JC (2021) Metagenome-assembled genomes: concepts, analogies, and challenges. Biophys Rev 13:905–909. 10.1007/s12551-021-00865-y 35059016 10.1007/s12551-021-00865-y PMC8724365

[CR95] Shen Z, Ruan Y, Chao X et al (2015) Rhizosphere microbial community manipulated by 2 years of consecutive biofertilizer application associated with banana *Fusarium* wilt disease suppression. Biol Fertil Soils 51:553–562. 10.1007/s00374-015-1002-7

[CR96] Shi H, Lu L, Ye J, Shi L (2022a) Effects of Two *Bacillus Velezensis* Microbial Inoculants on the Growth and Rhizosphere Soil Environment of *Prunus davidiana*. Int J Mol Sci 23:13639. 10.3390/ijms232113639 36362427 10.3390/ijms232113639 PMC9657632

[CR97] Shi J-W, Lu L-X, Shi H-M, Ye J-R (2022b) Effects of Plant Growth-Promoting Rhizobacteria on the Growth and Soil Microbial Community of *Carya illinoinensis*. Curr Microbiol 79:352. 10.1007/s00284-022-03027-9 36209341 10.1007/s00284-022-03027-9

[CR98] Shi L, Du N, Shu S et al (2017) *Paenibacillus polymyxa* NSY50 suppresses *Fusarium* wilt in cucumbers by regulating the rhizospheric microbial community. Sci Rep 7:41234. 10.1038/srep41234 28198807 10.1038/srep41234 PMC5304210

[CR99] Sibanyoni NR, Mmotla K, Mashabela MD et al (2026) Chemical dialogues in the rhizosphere: Metabolomics perspectives on plant defence and microbial interactions. Plant Soil 518:577–603. 10.1007/s11104-025-08010-4

[CR100] Simas DLR, Chagas FO, Da Silva AJR (2025) Plant–pathogen interaction: chemical mediation in plant signalling and defence. Phytochem Rev 24:5729–5745. 10.1007/s11101-025-10091-z

[CR101] Singh P, Chauhan PK, Upadhyay SK et al (2022) Mechanistic Insights and Potential Use of Siderophores Producing Microbes in Rhizosphere for Mitigation of Stress in Plants Grown in Degraded Land. Front Microbiol 13:898979. 10.3389/fmicb.2022.898979 35898908 10.3389/fmicb.2022.898979 PMC9309559

[CR102] Song J, Kong Z-Q, Zhang D-D et al (2021) Rhizosphere Microbiomes of Potato Cultivated under *Bacillus subtilis* Treatment Influence the Quality of Potato Tubers. Int J Mol Sci 22:12065. 10.3390/ijms222112065 34769506 10.3390/ijms222112065 PMC8584837

[CR103] Sun X, Xu Z, Xie J et al (2022) *Bacillus velezensis* stimulates resident rhizosphere *Pseudomonas stutzeri* for plant health through metabolic interactions. ISME J 16:774–787. 10.1038/s41396-021-01125-3 34593997 10.1038/s41396-021-01125-3 PMC8483172

[CR104] Tao C, Li R, Xiong W et al (2020) Bio-organic fertilizers stimulate indigenous soil *Pseudomonas* populations to enhance plant disease suppression. Microbiome 8:137. 10.1186/s40168-020-00892-z 32962766 10.1186/s40168-020-00892-z PMC7510105

[CR105] Tariq H, Subramanian S, Geitmann A, Smith DL (2025) *Bacillus* and *Paenibacillus* as plant growth-promoting bacteria in soybean and cannabis. Front Plant Sci 16:1529859. 10.3389/fpls.2025.1529859 40525084 10.3389/fpls.2025.1529859 PMC12169014

[CR106] Tripathi S, Srivastava P, Devi RS, Bhadouria R (2020) Influence of synthetic fertilizers and pesticides on soil health and soil microbiology. In: Prasad MNV (ed) Agrochemicals detection, treatment and remediation. Elsevier, pp 25–54

[CR107] Veselova MA, Plyuta VA, Khmel IA (2019) Volatile Compounds of Bacterial Origin: Structure, Biosynthesis, and Biological Activity. Microbiology 88:261–274. 10.1134/S0026261719030160

[CR108] Vuolo F, Novello G, Bona E et al (2022) Impact of Plant-Beneficial Bacterial Inocula on the Resident Bacteriome: Current Knowledge and Future Perspectives. Microorganisms 10:2462. 10.3390/microorganisms10122462 36557714 10.3390/microorganisms10122462 PMC9781654

[CR109] Wang H, Wu Q, Wang H et al (2025) The hidden diversity and functional potential of Chloroflexota genomes in arsenic and antimony co-contaminated soils. Soil Ecol Lett 7:240266. 10.1007/s42832-024-0266-y

[CR110] Wang J, Xu S, Yang R et al (2021) *Bacillus amyloliquefaciens* FH-1 significantly affects cucumber seedlings and the rhizosphere bacterial community but not soil. Sci Rep 11:12055. 10.1038/s41598-021-91399-6 34103586 10.1038/s41598-021-91399-6 PMC8187646

[CR111] Wang J, Zhao S, Xu S et al (2023) Co-inoculation of antagonistic *Bacillus velezensis* FH-1 and *Brevundimonas diminuta* NYM3 promotes rice growth by regulating the structure and nitrification function of rhizosphere microbiome. Front Microbiol 14:1101773. 10.3389/fmicb.2023.1101773 36846752 10.3389/fmicb.2023.1101773 PMC9948033

[CR112] Wang Z, Yang T, Mei X et al (2022) Bio-Organic Fertilizer Promotes Pear Yield by Shaping the Rhizosphere Microbiome Composition and Functions. Microbiol Spectr 10:e03572–e03522. 10.1128/spectrum.03572-22 36453930 10.1128/spectrum.03572-22 PMC9769518

[CR113] Wani PA, Khan MS, Zaidi A (2007) Chromium Reduction, Plant Growth–Promoting Potentials, and Metal Solubilizatrion by *Bacillus* sp. Isolated from Alluvial Soil. Curr Microbiol 54:237–243. 10.1007/s00284-006-0451-5 17294325 10.1007/s00284-006-0451-5

[CR114] Win KT, Okazaki K, Ohkama-Ohtsu N et al (2020) Short-term effects of biochar and *Bacillus pumilus* TUAT-1 on the growth of forage rice and its associated soil microbial community and soil properties. Biol Fertil Soils 56:481–497. 10.1007/s00374-020-01448-x

[CR115] Xiang H, He Y, Wang X et al (2023) Identification and characterization of siderophilic biocontrol strain SL-44 combined with whole genome. Environ Sci Pollut Res 30:62104–62120. 10.1007/s11356-023-26272-2 10.1007/s11356-023-26272-2 36940032

[CR116] Xu J, Qin L, Xu X et al (2023) *Bacillus paralicheniformis* RP01 Enhances the Expression of Growth-Related Genes in Cotton and Promotes Plant Growth by Altering Microbiota inside and outside the Root. Int J Mol Sci 24:7227. 10.3390/ijms24087227 37108389 10.3390/ijms24087227 PMC10138817

[CR117] Xu R, Liu H, Chen Y et al (2025) Responses of Rhizospheric Microbial Communities to *Brevibacillus laterosporus*-Enhanced Reductive Soil Disinfestation in Continuous Cropping Systems. Agronomy 15:1775. 10.3390/agronomy15081775

[CR118] Yang L, Qian X, Zhao Z et al (2024a) Mechanisms of rhizosphere plant-microbe interactions: molecular insights into microbial colonization. Front Plant Sci 15:1491495. 10.3389/fpls.2024.1491495 39606666 10.3389/fpls.2024.1491495 PMC11600982

[CR119] Yang P, Condrich A, Scranton S et al (2024b) Utilizing Plant Growth-Promoting Rhizobacteria (PGPR) to Advance Sustainable Agriculture. Bacteria 3:434–451. 10.3390/bacteria3040030

[CR120] Yannarell SM, Grandchamp GM, Chen S-Y et al (2019) A Dual-Species Biofilm with Emergent Mechanical and Protective Properties. J Bacteriol 201. 10.1128/JB.00670-18 10.1128/JB.00670-18 PMC6707914 30833350

[CR121] Ye S, Ma Y, Zhou S et al (2024) Biocontrol potential of *Priestia megaterium* YB-3 against *Meloidogyne graminicola* and its impact on the rhizosphere microbial community. J Pest Sci 97:2237–2256. 10.1007/s10340-024-01753-6

[CR122] Yu M, He H, Cheng L et al (2025) Bio-Organic Fertilizers Enhance Yield in Continuous Cotton Cropping Systems Through Rhizosphere Microbiota Modulation and Soil Nutrient Improvement. Agronomy 15:2238. 10.3390/agronomy15092238

[CR123] Zhang M-T, Bao Y-Q, Feng B-Y et al (2024) Impact of a Potent Strain of Plant Growth-Promoting Bacteria (PGPB), *Bacillus subtilis* S1 on Bacterial Community Composition, Enzymatic Activity, and Nitrogen Content in Cucumber Rhizosphere Soils. Curr Microbiol 81:358. 10.1007/s00284-024-03876-6 39285060 10.1007/s00284-024-03876-6

[CR124] Zhang Y, Gao X, Shen Z et al (2019) Pre-colonization of PGPR triggers rhizosphere microbiota succession associated with crop yield enhancement. Plant Soil 439:553–567. 10.1007/s11104-019-04055-4

[CR125] Zhang Z, Claessen D, Rozen DE (2016) Understanding Microbial Divisions of Labor. Front Microbiol. 10.3389/fmicb.2016.02070. 7:2070 28066387 10.3389/fmicb.2016.02070 PMC5174093

[CR126] Zhao W, Ban Y, Su Z et al (2023) Colonization Ability of *Bacillus subtilis* NCD-2 in Different Crops and Its Effect on Rhizosphere Microorganisms. Microorganisms 11:776. 10.3390/microorganisms11030776 36985349 10.3390/microorganisms11030776 PMC10058285

[CR127] Zhou S, Liu G, Zheng R et al (2020) Structural and Functional Insights into Iturin W, a Novel Lipopeptide Produced by the Deep-Sea Bacterium *Bacillus* sp. Strain wsm-1. Appl Environ Microbiol 86:e01597–e01520. 10.1128/AEM.01597-20 32859591 10.1128/AEM.01597-20 PMC7580537

[CR128] Zhu Q, Zhou J, Sun M et al (2023) A newly isolated *Bacillus megaterium* OQ560352 promotes maize growth in saline soils by altering rhizosphere microbial communities and organic phosphorus utilization. Rhizosphere 27:100746. 10.1016/j.rhisph.2023.100746

[CR129] Zuluaga MYA, Milani KML, Miras-Moreno B et al (2021) Inoculation with plant growth-promoting bacteria alters the rhizosphere functioning of tomato plants. Appl Soil Ecol 158:103784. 10.1016/j.apsoil.2020.103784

